# Rab11 Regulates Trafficking of *Trans*-sialidase to the Plasma Membrane through the Contractile Vacuole Complex of *Trypanosoma cruzi*

**DOI:** 10.1371/journal.ppat.1004224

**Published:** 2014-06-26

**Authors:** Sayantanee Niyogi, Juan Mucci, Oscar Campetella, Roberto Docampo

**Affiliations:** 1 Department of Cellular Biology and Center for Tropical and Emerging Global Diseases, University of Georgia, Athens, Georgia, United States of America; 2 Instituto de Investigaciones Biotecnológicas, Universidad Nacional de San Martín/Consejo Nacional de Investigaciones Científicas y Técnicas, Buenos Aires, Argentina; National Institutes of Health, United States of America

## Abstract

*Trypanosoma cruzi* is the etiologic agent of Chagas disease. Although this is not a free-living organism it has conserved a contractile vacuole complex (CVC) to regulate its osmolarity. This obligate intracellular pathogen is, in addition, dependent on surface proteins to invade its hosts. Here we used a combination of genetic and biochemical approaches to delineate the contribution of the CVC to the traffic of glycosylphosphatidylinositol (GPI)-anchored proteins to the plasma membrane of the parasite and promote host invasion. While *T. cruzi* Rab11 (GFP-TcRab11) localized to the CVC, a dominant negative (DN) mutant tagged with GFP (GFP-TcRab11DN) localized to the cytosol, and epimastigotes expressing this mutant were less responsive to hyposmotic and hyperosmotic stress. Mutant parasites were still able to differentiate into metacyclic forms and infect host cells. GPI-anchored *trans*-sialidase (TcTS), mucins of the 60–200 KDa family, and trypomastigote small surface antigen (TcTSSA II) co-localized with GFP-TcRab11 to the CVC during transformation of intracellular amastigotes into trypomastigotes. Mucins of the gp35/50 family also co-localized with the CVC during metacyclogenesis. Parasites expressing GFP-TcRab11DN prevented TcTS, but not other membrane proteins, from reaching the plasma membrane, and were less infective as compared to wild type cells. Incubation of these mutants in the presence of exogenous recombinant active, but not inactive, TcTS, and a sialic acid donor, before infecting host cells, partially rescued infectivity of trypomastigotes. Taking together these results reveal roles of TcRab11 in osmoregulation and trafficking of *trans*-sialidase to the plasma membrane, the role of *trans*-sialidase in promoting infection, and a novel unconventional mechanism of GPI-anchored protein secretion.

## Author Summary

Several free-living protozoa possess a contractile vacuole complex (CVC) that protects them from the hyposmotic environments where they live. Interestingly, the intracellular parasite *Trypanosoma cruzi*, the etiologic agent of Chagas disease, has conserved a CVC in all its developmental stages, where it has an osmoregulatory role under both hyposmotic and hyperosmotic conditions. We found here that the CVC of *T. cruzi* has an additional unconventional role in traffic of glycosylphosphatidylinositol (GPI)-anchored proteins to the plasma membrane of the parasite. A combination of genetic and biochemical approaches revealed the role of TcRab11, a protein localized to the CVC, in traffic of *trans*-sialidase (TcTS), a GPI-anchored protein important for host cell invasion, but not of other GPI-anchored proteins or integral membrane proteins, to the plasma membrane. Demonstration of the role of TcTS in infection has been previously difficult given the large number of genes encoding for this protein distributed through the genome of the parasite. However, by constructing dominant negative TcRab11 we were able to prevent traffic of TcTS to the plasma membrane and demonstrate its role in host invasion.

## Introduction

The contractile vacuole complex (CVC) is an intracellular compartment with an osmoregulatory role in different protists. This compartment has a bipartite structure, consisting of a central vacuole or bladder and a surrounding loose network of tubules and vesicles named the spongiome [1], [2]. The CVC accumulates water through an aquaporin [3]–[7] and expels it out of the cell through pores in the plasma membrane [1], [2]. *Trypanosoma cruzi*, the etiologic agent of Chagas disease or American trypanosomiasis, possesses a CVC [4], [8], [9] that is important for regulatory volume decrease (RVD) after hyposmotic stress [4], as well as for shrinking of the cells when submitted to hyperosmotic stress [10].

Besides its osmoregulatory role, the CVC of some protists is an acidic calcium store [11] and has roles in calcium ion (Ca^2+^) sequestration and excretion pathways [12]–[16], as well as in transfer of some proteins to the plasma membrane [12], [17], [18]. Although it has been indicated that there is no much mixing or “scrambling” of contractile vacuoles and plasma membranes [19], transfer of membrane proteins from the CVC to the plasma membrane has been observed. In *Dictyostelium discoideum*, the vacuolar proton ATPase (V-H^+^-ATPase) and calmodulin (CaM) move to the plasma membrane when cells are starved during stationary phase [17], and the Ca^2+^-ATPase PAT1 moves to the plasma membrane when cells are incubated at high Ca^2+^ concentrations [12]. Some luminal proteins, such as the adhesins DdCAD-1 and discoidin-1 can also be targeted to the cell surface via the CVC in *D. discoideum* [18], [20]. In *T. cruzi* epimastigotes, the polyamine transporter TcPOT1.1, which localizes to CVC-like structures, has also been reported to appear in the plasma membrane when the culture medium is deficient in polyamines [21]. It is interesting to note that dajumin-GFP is trafficked to the CVC of *D. discoideum* via the plasma membrane and is internalized by a clathrin-dependent mechanism, suggesting that clathrin-mediated endocytosis may function as a back-up mechanism in case of transfer of proteins from the CVC to the plasma membrane [22].

It is remarkable that Rab11, a GTPase that localizes in recycling endosomes in most cells [23], including *Trypanosoma brucei* [24], localizes to the CVC of *D. discoideum* [25] and *T. cruzi* [26], suggesting that it might have some function in trafficking of proteins from the CVC to the plasma membrane, as recycling endosomes have. It was proposed [25] that the CVC could be an evolutionary precursor to the recycling endosomal system in other eukaryotes.

In *T. brucei*, Rab11 mediates the transfer of the glycosylphosphatidylinositol (GPI)-anchored proteins transferrin [27] and variant surface glycoprotein (VSG) [28] to the plasma membrane. *T. cruzi* is also rich in GPI-anchored proteins, among them the *trans*-sialidase (TS)-like superfamily, which includes 1,430 gene members [29], [30], and the mucins, encoded by 500 to 700 genes distributed into three groups of which group III is conformed by a single-copy gene named the trypomastigote small surface antigen (*TSSA*) [31]. *TcTS* genes are actually distributed in several families of which only one is composed by genes encoding the active enzyme (TS) and its inactive isoform (iTS), which differs in only one mutation (Tyr342His) [32]. TcTS is crucial in the life cycle of the parasite because it allows the acquisition of sialyl residues from the host glycoconjugates preventing their lysis by the alternative complement pathway [33], [34], and opsonization followed by killing by natural antibodies [35]. It also enables the parasite to infect/attach cells [36], [37], and exit the parasitophorous vacuole [38]. The shed TcTS induces several hematological abnormalities and alters the immune system [39]–[41]. Two major TcTSSA isoforms were originally recognized: TcTSSA I, present in TcI parasite stocks, which are linked to the sylvatic cycle of the parasite, and TcTSSA II, present in TcVI (previously TcIIe) isolates, which are linked to the more virulent strains [31]. Since TcTSSA II is highly immunogenic it has been proposed as an immunological marker for the most virulent *T. cruzi* types [31], and as an adhesin, engaging surface receptor(s) and inducing signaling pathways in the host cell as a prerequisite for parasite internalization [42]. Another group of GPI-anchored surface proteins is that formed by the mucin family of 60–200 KDa proteins bearing several oligosaccharide chains and present in tissue culture-derived trypomastigotes [43]. These *T. cruzi* O-linked oligosaccharide-containing proteins are highly immunogenic under the conditions of natural infection and are the targets for lytic anti-Gal antibodies [43]–[45]. Gp35/50 mucins are also GPI-anchored glycoproteins rich in threonine and expressed in epimastigotes and metacyclic forms of all *T. cruzi* isolates examined to date and are encoded by a large multigene family [46]. Gp35/50 mucins are recognized by monoclonal antibodies 10D8 and 2B10 [47], which react with galactofuranose- and galactopyranose-containing epitopes, respectively.

GPI-anchored proteins are usually transported from the endoplasmic reticulum (ER) to the plasma membrane through the Golgi apparatus, where lipid raft-like structures form [48]. In this work we demonstrate that TcTS, TcTSSA II, and other mucins are transported to the plasma membrane of *T. cruzi* trypomastigotes through the CVC, which also possesses lipid-raft like structures, and that expression of dominant-interfering TcRab11 mutants altered their morphology, osmoregulation, traffic of TcTS to the plasma membrane, and parasite infectivity. The results suggest the presence of a novel unconventional mechanism of GPI-anchored protein transport to the cell surface of eukaryotic cells.

## Results

### Localization of TcRab11 in different *T. cruzi* stages

**Figure 1:**
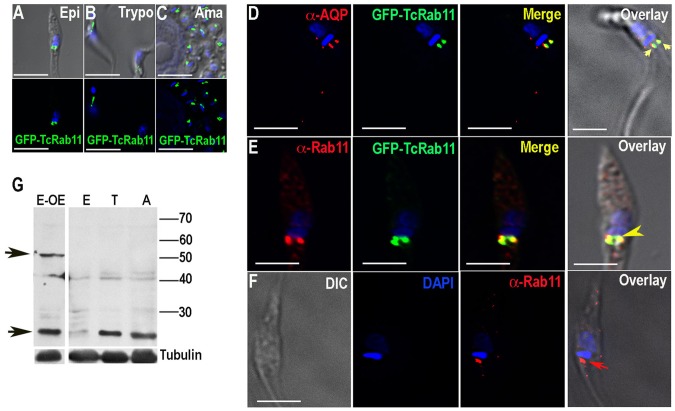
Fluorescence microscopy analysis of TcRab11 in different stages of *T. cruzi*. (A–C) GFP fusion protein of TcRab11 was detected in the contractile vacuole bladder of epimastigotes (Epi, A), trypomastigotes (Trypo, B), and intracellular amastigotes (Ama, C) using antibodies against GFP. Upper panels show differential interference contrast microscopy (DIC) images merged with DAPI staining of DNA (in *blue*) and GFP-TcRab11 (in *green*). Lower panels show fluorescence images. (D) GFP-TcRab11 (*green*) co-localizes with antibodies against *T. cruzi* aquaporin 1 (α-AQP, *red*), a marker for the contractile vacuole, under hyposmotic conditions. (E) Antibodies against TbRab11 (α-Rab11, *red*) co-localize with GFP-TcRab11 (*green*). (F) Antibodies against TbRab11 (*red*) localize to a compartment that resembles the contractile vacuole in (E). DAPI staining is in *blue*. Arrowheads in D–F show co-localization between antibodies against TcAQP1 and GFP (D), TbRab11 antibody and GFP (E) and labeling with antibodies against TbRab11 (F), respectively. Bars in A–F = 10 µm. (G) Western blot analyses with TbRab11 antibody of lysates of epimastigotes overexpressing GFP-TcRab11 (E-OE), or wild-type epimastigotes (E), trypomastigotes (T) and amastigotes (A) showing bands (*arrows*) corresponding to the endogenous TcRab11 (24 kDa) and to GFP-TcRab11 (50 kDa). The blots were sequentially probed with αTbRab11 and anti-tubulin antibodies, used as loading control.

In previous work we reported the N-terminal tagging of *T. cruzi Rab11* (TcCLB.511407.60; *TcRab11*) with the green fluorescent protein (*GFP*) gene, and the localization of GFP-TcRab11 to the bladder of the CVC of epimastigotes of *T. cruzi* [26]. Tagging with GFP was confirmed by western blot analysis [26]. Fig. 1A–C shows now that GFP-TcRab11 localizes to the bladder of the CVC of epimastigotes, trypomastigotes, and amastigotes. Fig. 1D shows the co-localization of GFP-TcRab11 with *T. cruzi* aquaporin 1 (TcAQP1), a marker for the CVC [3], [4]. These experiments were done after submitting the cells to hyposmotic conditions, which increases the localization of TcAQP1 to the CVC [4]. To confirm that the above results were not an artifact of protein overexpression and/or mistargeting we also used affinity-purified antibodies against TbRab11 [24] (Fig. 1E and 1F). This antibody was shown to predominantly react with a protein of 24 kDa in all *T. cruzi* stages, as expected for TcRab11 (Fig. 1G). TcRab11 is apparently less expressed in epimastigotes. Fig. S1A–D confirms the CVC localization of GFP-TcRab11 in epimastigotes submitted to hyposmotic stress by cryo-immunogold electron microscopy.

### Localization of GFP-TcRab11DN mutant

**Figure 2:**
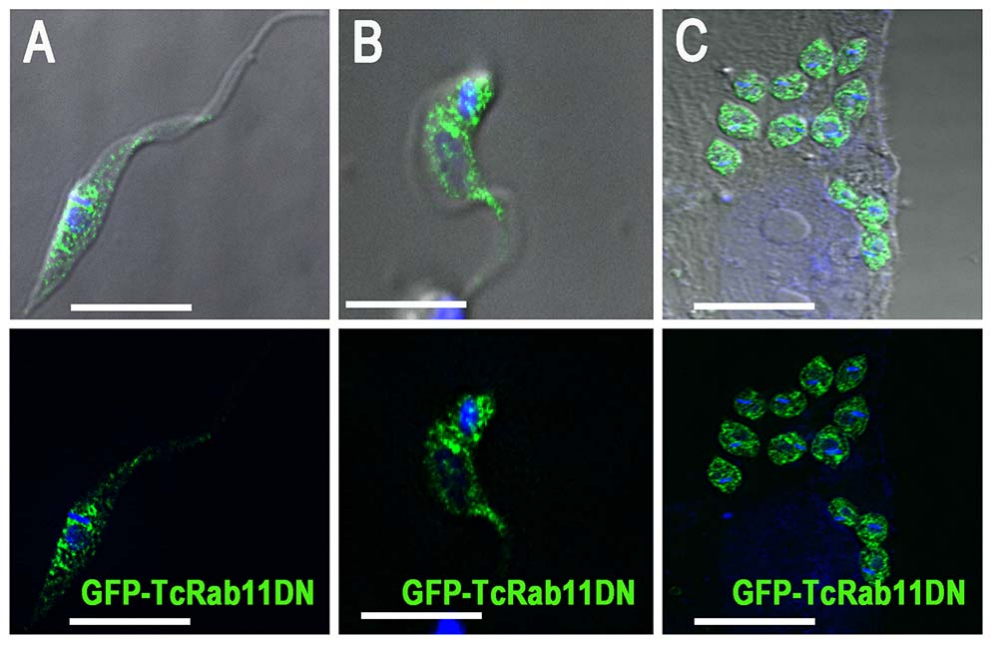
GFP-TcRab11DN localizes to the cytoplasm of different life cycle stages. (A–C) GFP-TcRab11DN, mimicking the GDP–bound state of the protein has a cytosolic punctate localization in epimastigotes, (A), trypomastigotes (B), and intracelular amastigotes (C), as detected using antibodies against GFP. DNA was stained with DAPI. Bars = 10 µm.

Knockdown of Rabs by RNA interference (RNAi) is one of the preferred approaches to investigate the function of specific Rab isoforms in membrane traffic [49]. Unfortunately, *T. cruzi* lacks an RNAi system [50]. To perform a functional analysis of TcRab11, we therefore developed an expression plasmid encoding a TcRab11 mutant that mimics the GDP-bound form (dominant negative). An N-terminal GFP epitope tag was fused to the *T. cruzi* point mutant TcRab11:S21N. TcRab11:S21N is predicted to bind GDP, based upon homology to known Ras-related protein mutations [51]. In transfected *T. cruzi* epimastigotes, GFP-TcRab11DN had a punctated cytosolic localization (Fig. 2A). This localization was maintained when epimastigotes were differentiated into trypomastigotes (Fig. 2B) and intracellular amastigotes (Fig. 2C). This localization is because the dominant negative TcRab11 (GDP-bound) gets locked in an intermediate cytosolic location. After membrane delivery by the GDP dissociation inhibitor (GDI), Rab proteins interconvert between inactive, GDP-bound forms and active, GTP-bound forms [52]. The growth rate of the mutant epimastigotes was not affected (Fig. S2A). We confirmed tagging of the mutant by western blot analysis (Fig. S2B). Together these results suggest that TcRab11 is localized to the membrane of the CVC in a GTP-dependent manner. Densitometry analysis indicated that GFP-TcRab11 expression increased 5.2 fold compared to that in wild type epimastigotes (Fig. S2C). We also investigated whether the dominant negative mutation of TcRab11 disrupted the structure and assembly of the CVC. We did immunofluorescence studies on GFP-TcRab11DN mutant epimastigotes using an antibody against *T. cruzi* aquaporin 1, a CVC marker [4]. The same aquaporin distribution was observed in epimastigotes expressing the control GFP-TcRab11 (Fig. S3A) and the mutant GFP-TcRab11DN (Fig. S3B). The CVC can be identified in Fig. S3A and S3B because of its curvature and its location close to the kinetoplast. There was a greater concentration of TcAQP1 in the CVC with some punctate labeling corresponding to acidocalcisomes [4] (Fig. S3).

### Cellular response to hyposmotic and hyperosmotic stresses

**Figure 3:**
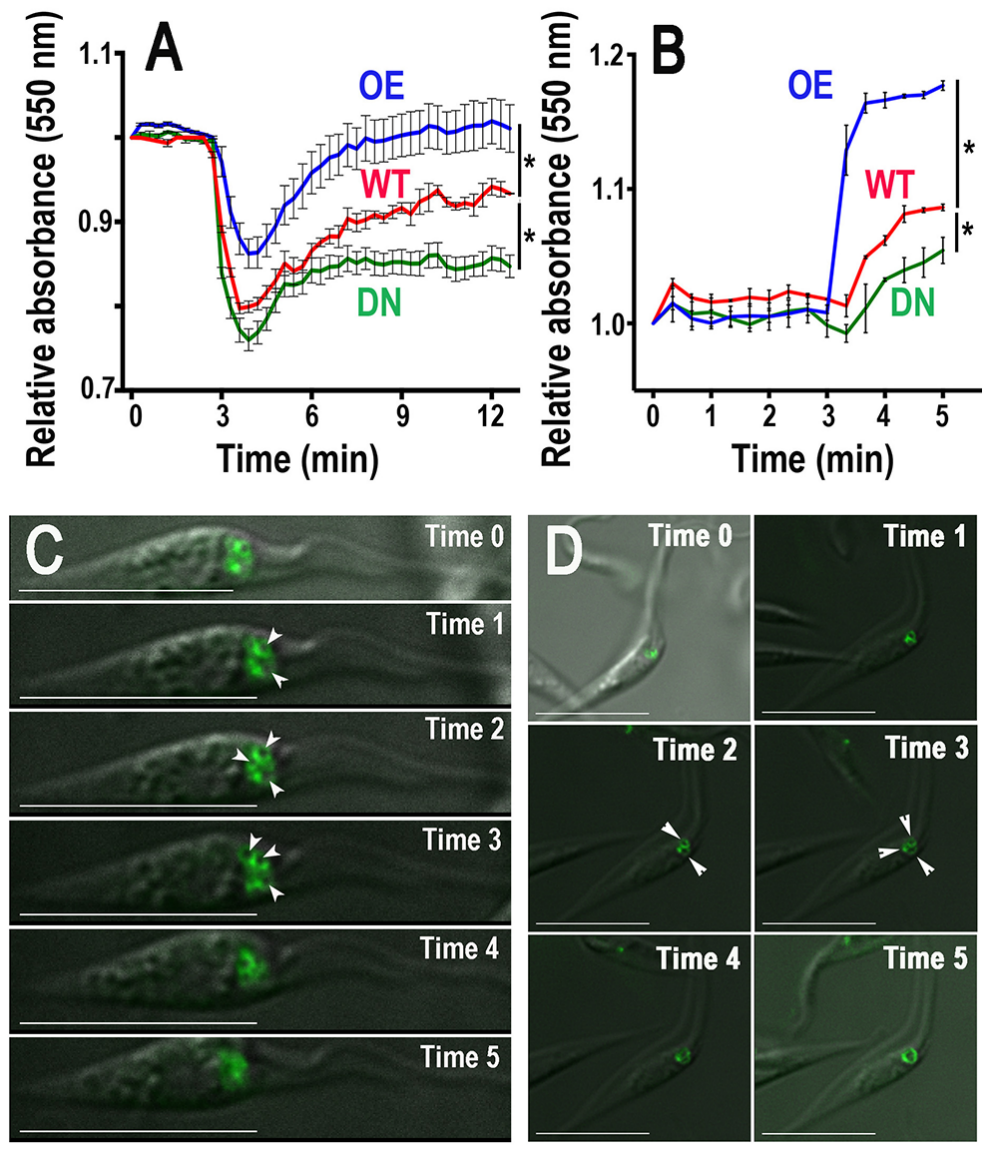
Regulatory volume changes of epimastigotes. (A–B) Cells were pre-incubated in isosmotic buffer for 3 min and then subjected to hyposmotic (final osmolarity = 150 mOsm) (A) or hyperosmotic (final osmolarity = 650 mOsm) (B) stress. Relative change in cell volume was followed by monitoring absorbance at 550 nm by light scattering. As compared to wild-type cells (WT), cells expressing GFP-TcRab11DN (DN) failed to fully recover their volume after hyposmotic stress and shrank less after hyperosmotic stress, while cells overexpressing GFP-TcRab11 (OE) recovered their volume faster after hyposmotic stress and shrank more after hyperosmotic stress. Values are means ± SD of three different experiments. Asterisks indicate statistically significant differences, *p*<0.05, (Bonferroni's multiple comparison “a posteriori” test of one-way ANOVA) at all time points after induction of osmotic stress. (C–D) Epimastigotes were immobilized on glass slides with poly-lysine and diluted with deionized water to a final osmolarity of 150 mOsm (C) or bathed with hyperosmotic (650 mOsm) buffer (D). Video microscopy data were collected and selected frames are shown. Times indicated in each frame represent 1 second apart after induction of stress. Arrowheads show different dilated compartments that transform into larger bladders at a later time. Results are representative of those obtained from at least three independent experiments. Bars = 10 µm.

To examine the role of *T. cruzi* Rab11 in osmoregulation, wild-type, GFP-TcRab11-overexpressing (GFP-TcRab11OE), and GFP-TcRab11DN-expressing epimastigotes were submitted to hyposmotic stress and their regulatory volume decrease (RVD) measured using the light-scattering technique, as described previously [53]. This technique measures the changes in volume of the cells under hyposmotic (swelling and recovery) and hyperosmotic conditions (shrinking and partial recovery). After recovery the cells recuperate their normal morphology. DN mutants were less able to recover their volume after hyposmotic stress than wild type cells, while recovery was faster in GFP-TcRab11OE cells (OE, Fig. 3A). In addition, when submitted to hyperosmotic stress, DN mutants shrank less while GFP-TcRab11OE cells shrank more than control cells (Fig. 3B), and in all cases they did not recover their volume during the time of the experiment. It has been shown previously that when epimastigotes are submitted to hyperosmotic stress the parasites do not regain their normal volume at least during the following two hours [10]. GFP-TcRab11OE epimastigotes were also studied under hyposmotic and hyperosmotic stress conditions by video fluorescence microscopy. Epimastigotes were immobilized on glass slides with poly-L-Lysine and bathed in hyposmotic/hyperosmotic buffer. Video microscopy data were collected (Videos S1 and S2; Figs. 3C and 3D show selected frames), which revealed changes in the morphology of the CVC when epimastigotes were treated under both hyposmotic (Fig. 3C, Video S1) and hyperosmotic (Fig. 3D, Video S2) conditions. The single fluorescent spot corresponding to the CVC could be seen enlarging and fusing with other vacuoles probably resulting from enlarged tubular structures of the spongiome. Altogether, these results confirm the active participation of the CVC on the cellular response to both hyposmotic and hyperosmotic stresses [10], and indicate that alteration of TcRab11 function leads to disruption of osmoregulatory processes.

### *Trans*-sialidase co-localizes with GFP-TcRab11 during differentiation of amastigotes into trypomastigotes

As Rab11 mediates the recycling of GPI-anchored proteins of *T. brucei* [27], [28] we investigated whether TcRab11 affected the traffic of GPI-anchored proteins in *T. cruzi*. *Trans*-sialidase is an abundant GPI-anchored protein present in the cell surface of trypomastigotes [54], [55], where it catalyzes the transfer of sialic acid from host proteins to parasite mucins [56].

**Figure 4:**
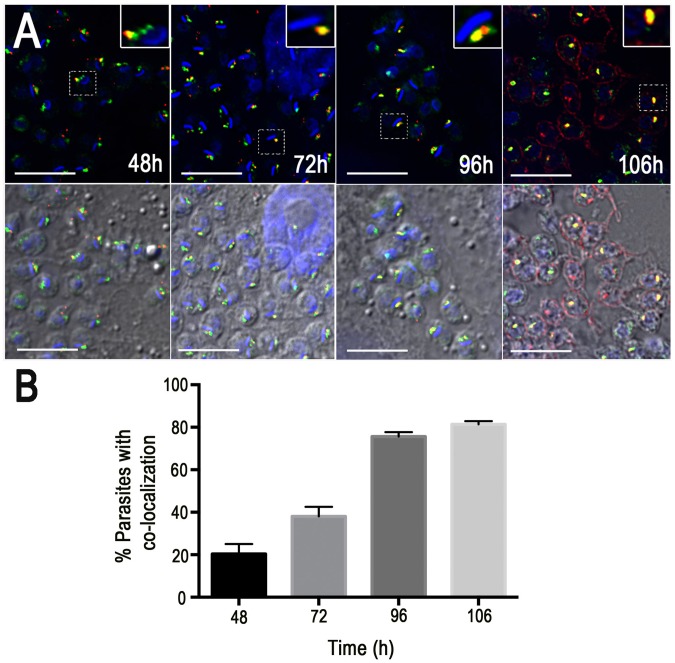
Co-localization of GFP-TcRab11 and TcTS during amastigote differentiation in human foreskin fibroblasts. (A) Expression of TcTS becomes apparent at 48 h p.i., when antibodies against TcTS (*red*) co-localize with GFP-TcRab11, as detected with antibodies against GFP (*green*). Co-localization progresses to close to 80% of cells by 96 h, and after 106 h co-localization starts to decrease and surface labeling of TcTS is more evident. Scale bars = 10 µm. Insets shows co-localization at high magnification (double). (B) Percentage of amastigotes showing co-localization of TcTS and GFP-Rab11 with time. Two hundred amastigotes were counted in each experiment and results are expressed as means ± SEM (n = 3).

**Figure 5:**
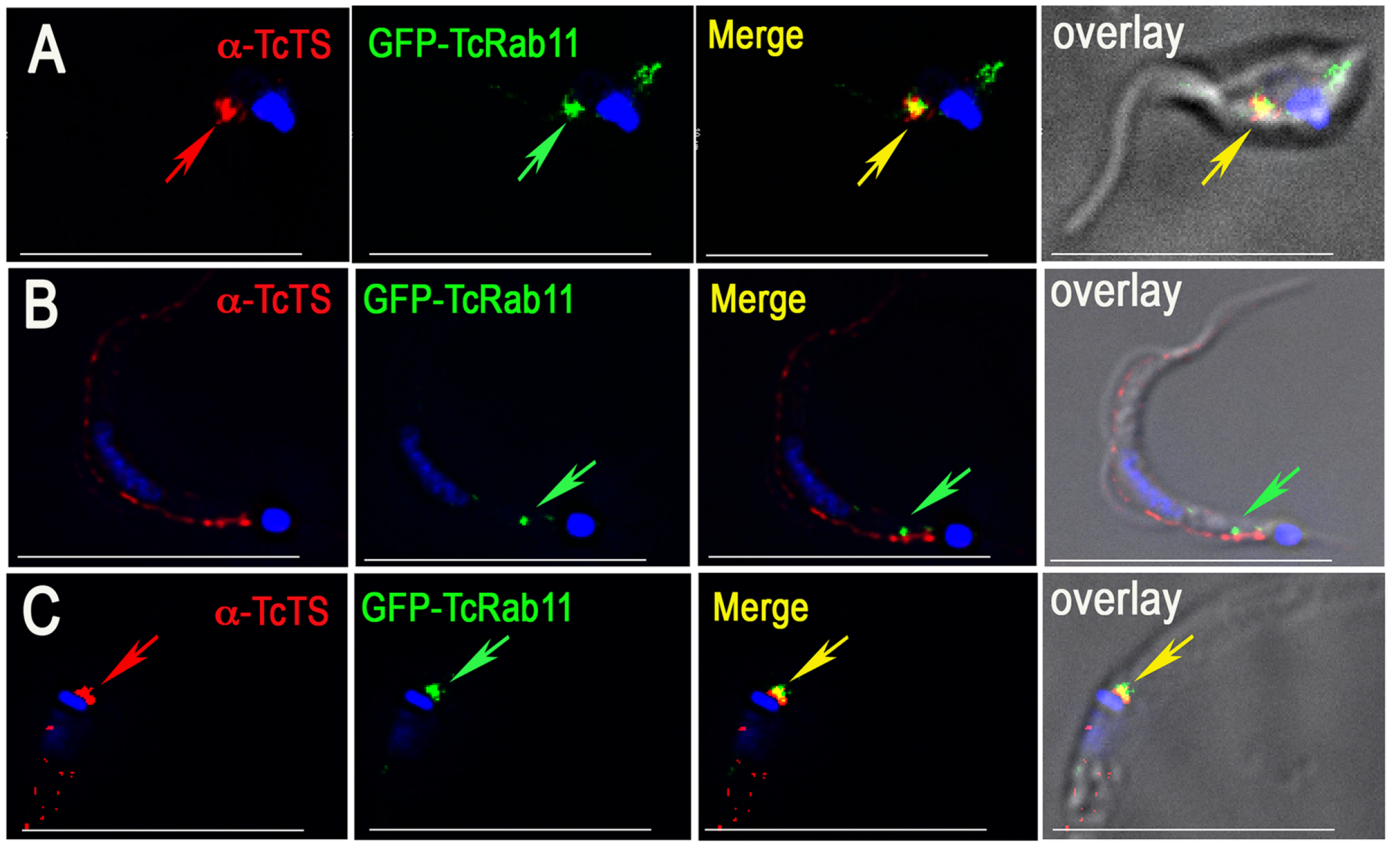
Localization of TcTS during differentiation to cell-derived and metacyclic trypomastigotes. (A) Co-localization of αTcTS and antibodies against GFP in intermediate stages (epimastigote-like) obtained from tissue culture supernatants. (B) TcTS localizes to patches of the plasma membrane in fully differentiated trypomastigotes while GFP-TcRab11 remains in the CVC. (C) Co-localization of αTcTS with GFP-TcRab11 in epimastigotes during transformation into metacyclic stages. Scale bars (C–E) = 10 µm.

To investigate the possibility that TcRab11 mediates the traffic of TcTS to the plasma membrane, we infected L_6_E_9_ myoblasts with metacyclic trypomastigotes from stationary cultures of GFP-TcRab11OE parasites and obtained cell culture-derived trypomastigotes. GFP-TcRab11OE trypomastigotes were used to infect fibroblasts and labeling of TcTS was detected by indirect immunofluorescence analysis using antibodies against the SAPA (shed acute phase antigen) repeats [57] at different time points during infection (Fig. 4A). We found reaction with these antibodies starting 48 h after infection when the reaction co-localized with GFP-TcRab11 in the contractile vacuole of intracellular amastigotes (Fig. 4A). Co-localization progressed to more than 80% of the cells by 106 h, after which, labeling of the CVC gradually disappeared and surface labeling was more evident (Figs. 4A, and 4B), suggesting that TcTS traffics through the contractile vacuole before reaching the plasma membrane in differentiating trypomastigotes. Intermediate stages between amastigotes and trypomastigotes (‘epimastigote-like’ forms) found in the supernatants of tissue culture cells also showed co-localization of GFP-TcRab11 and TS (Fig. 5A) but in fully differentiated trypomastigotes labeling of TcTS was predominantly in patches of the plasma membrane while GFP-TcRab11 labeling remained in the CVC (Fig. 5B).

**Figure 6:**
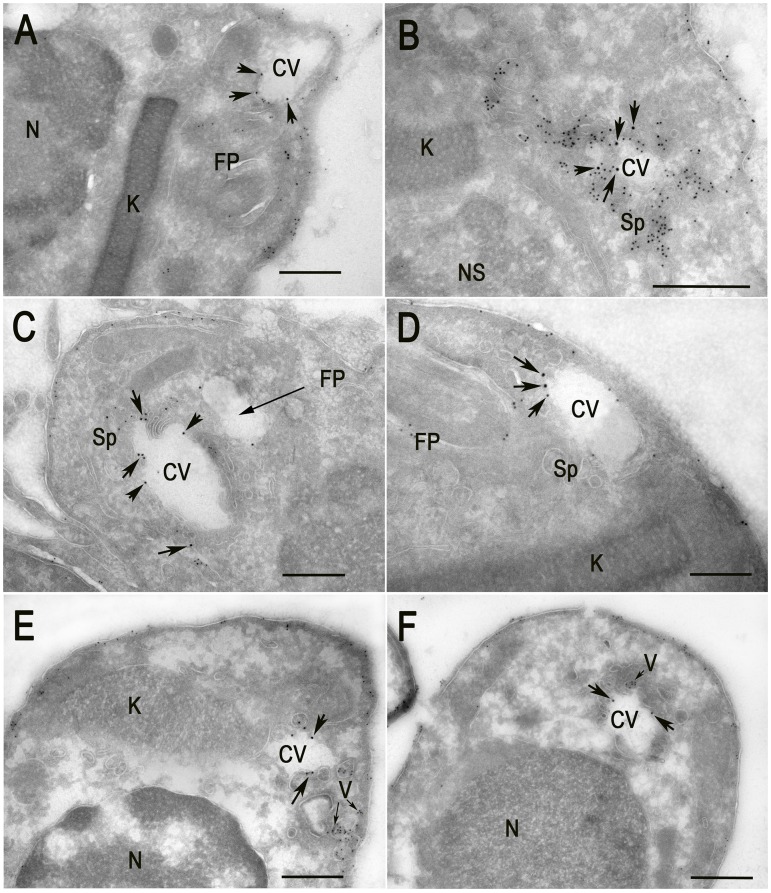
Cryo-immunoelectron microscopy localization of GFP-TcRab11 and TcTS in amastigotes. Amastigotes were isolated from HFF at different times p.i., as described under Materials and Methods. GFP-TcRab11 and TcTS were detected with goat anti-GFP, and rabbit anti-TcTS antibodies, and donkey anti-goat 18 nm colloidal gold and donkey anti-rabbit 12 nm colloidal gold, respectively. (A–D) Amastigotes obtained after 96 h p.i. Co-localization of antibodies against GFP (*arrows*) and TcTS (*small dots*) is evident in the CV bladder (CV) and spongiome (Sp), while TcTS also localizes to the flagellar pocket (FP) and in patches of the plasma membrane. Note in (B) a collapsed bladder and intense labeling of the spongiome. (E–F) Amastigotes obtained 106 h p.i. GFP-TcRab11 localizes to the CV bladder while TcTS localizes to vesicles (V, *small arrows*) close to the plasma membrane and in patches in the plasma membrane. Scale bars = 500 nm. Note that the patchy appearance of the cytoplasm is due to the absence of glutaraldehyde in the fixative because it abolished labeling of TcTS.

Cryo-immunogold electron microscopy confirmed the co-localization of GFP-TcRab11 and TcTS in the CVC (Fig. 6A). Co-localization was very intense in the spongiome of collapsed vacuoles (Fig. 6B). TcTS was also observed in the flagellar pocket (Figs. 6C, and D) and in patches in the plasma membrane (Figs. 6A, D), at earlier time points than by IFA analysis. At later time points stronger labeling of TcTS was detected in patches of the plasma membrane and in vesicles close to the surface (Figs. 6E, F). The surface localization of TcTS in trypomastigotes has been established before by immunogold electron microscopy studies [55], [58].

### *Trans*-sialidase co-localizes with GFP-TcRab11 in intermediate stages of differentiation from epimastigotes to metacylic trypomastigotes

As TcTS is also present in the surface of metacyclic trypomastigotes we investigated whether there was co-localization of TcTS with GFP-TcRab11 during differentiation of epimastigotes into metacyclic trypomastigotes as described under Materials and Methods. Fig. 5C shows the co-localization of antibodies against TcTS with GFP-TcRab11 in intermediate forms that appeared around day 5 of the metacyclogenesis process.

### TcRab11DN mutant prevents plasma membrane localization of TcTS but not of other plasma membrane proteins

To investigate whether mutation of TcRab11 affects general traffic of membrane proteins to the cell surface of trypomastigotes, wild type and GFP-TcRab11DN trypomastigotes were used to infect HF fibroblasts and labeling of TcTS and other membrane proteins were detected by indirect immmunofluorescence analysis after a full cycle of differentiation into trypomastigotes.

**Figure 7:**
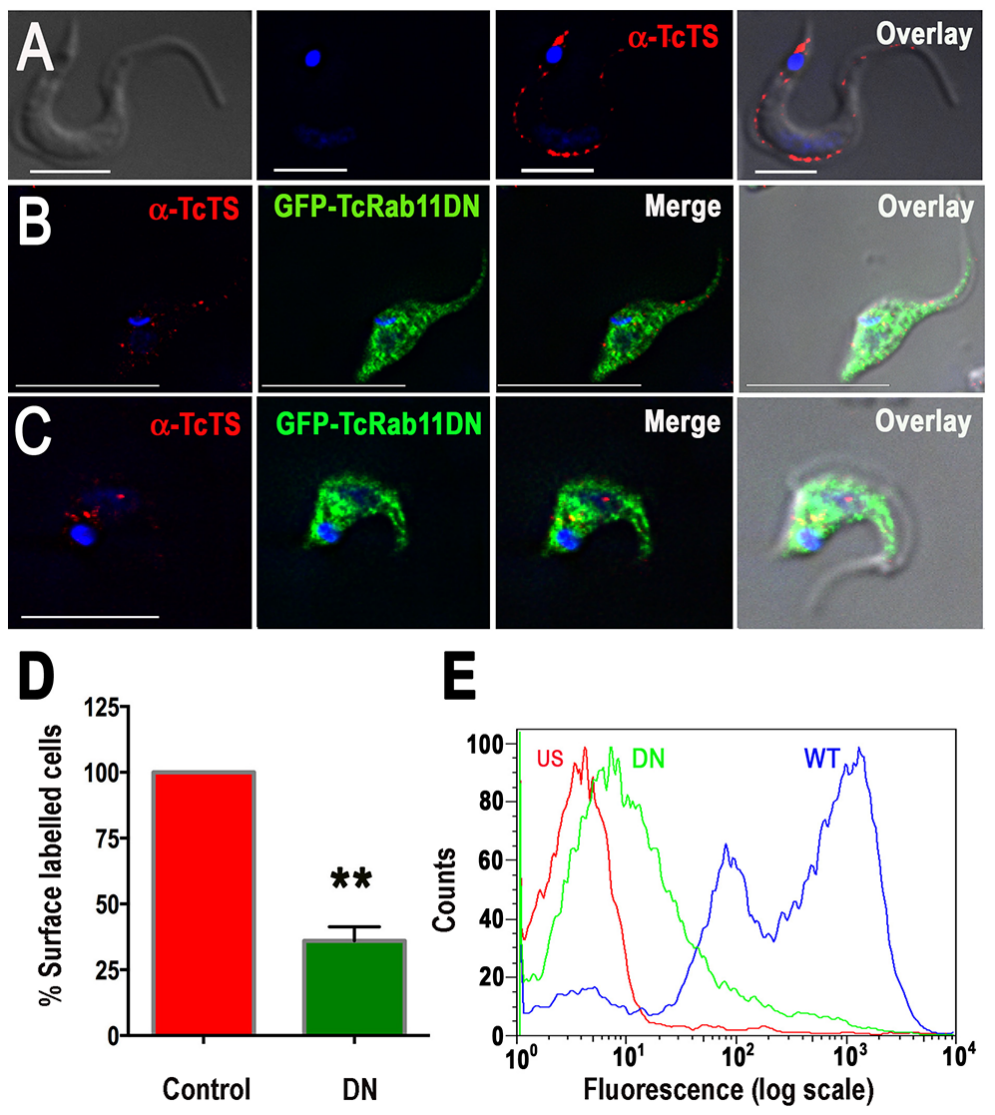
Overexpression of GFP-TcRab11DN reduces the surface expression of TcTS. Tissue culture-derived wild type, and GFP-TcRab11DN-expressing trypomastigotes and intermediate forms were fixed, permeabilized and stained with antibodies against TcTS (A), or both TcTS and GFP (B and C). Labeling of TcTS (*red*) in fully differentiated trypomastigotes was predominantly in surface patches (A). Labeling of GFP-TcRab11DN (*green*) was predominantly cytosolic while labeling of TcTS was punctated but did not reach the cell surface in intermediate forms (B) or fully differentiated trypomastigotes (C). (D) The fluorescence intensity of TcTS in the cell surface of tissue culture-derived GFP-TcRab11DN-expressing trypomastigotes was measured in 200 cells in each experiment and expressed as percentage of control (wild-type trypomastigotes). Values are means ± SEM of 3 independent experiments. **p<0.05. (E) FACS analysis of fixed GFP-TcRab11DN trypomastigotes reveals a decrease in the surface expression of TcTS as depicted by their lesser fluorescence intensity (DN) in comparison to that of wild type cells (WT). The negative control were unstained wild type trypomastigotes (US) showing background fluorescence. Wild type cells have two peaks of TcTS, suggesting the presence of intermediate stages in these asynchronously growing cultures. Data is representative of the profile analysis of 20,000 cells from 3 independent experiments.

Wild type trypomastigotes showed labeling of TcTS in the plasma membrane (Fig. 7A) while GFP-TcRab11DN intermediate forms (Fig. 7B) and trypomastigotes (Fig. 7C), identified by the position of the kinetoplast anterior or posterior to the nucleus, respectively, showed predominantly cytosolic labeling of TcTS (Fig. 7B–D). This weak intracellular label with TcTS could be the result of ER retention and export to the cytosol that ultimately results in its degradation by the ubiquitin/proteasome system [59]. Labeling of GFP-TcRab11DN was predominantly punctated cytosolic, as described above for epimastigotes (Fig. 2A). These results suggest that DN mutation of TcRab11 inhibits traffic of TcTS to the plasma membrane. To further confirm this observation we used SAPA antibodies to assess surface expression of TcTS by flow cytometry on GFP-TcRab11DN and wild type trypomastigotes. As expected, flow cytometric analysis shows reduction in surface expression of TcTS in the mutants as compared to control wild type trypomastigotes (Fig. 7E). Western blot analyses showed that these trypomastigotes maintained the overexpression of GFP-TcRab11 and GFP-TcRab11DN (Fig. S2D). To address the specificity of this TcTS antibody, total parasite lysates of wild type and GFP-TcRab11DN were subjected to western blot analyses. Signals were observed in both lanes, matching the expected size of the TcTSs [37], [60] (Fig. S3C).

**Figure 8:**
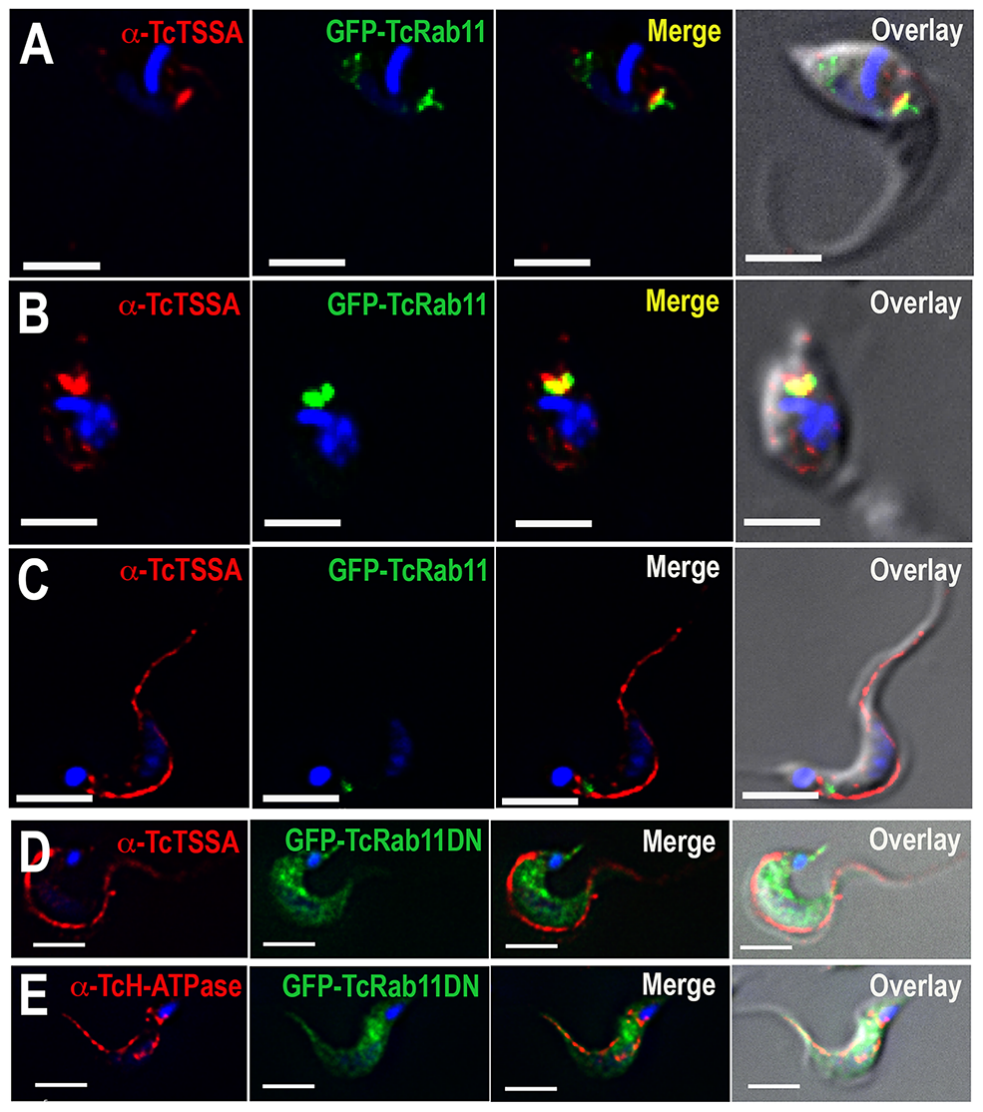
Localization of surface proteins in GFP-TcRab11OE and GFP-TcRab11DN-expressing parasites. Antibodies against TcTSSA II (*red*) co-localize with antibodies against GFP (*green*) in intermediate forms (A) and amastigotes (B) but not in trypomastigotes expressing GFP-TcRab11, where they localize to the plasma membrane (C). Antibodies against TcTSSA II (D) still localize to the plasma membrane in GFP-TcRab11DN-expressing cells, while antibodies against the H^+^-ATPase (E) maintain their intracellular and plasma membrane localization in GFP-Rab11DN-expressing cells. In (D) and (E) GFP staining localizes to the cytosol. Scale bars = 10 µm.

**Figure 9:**
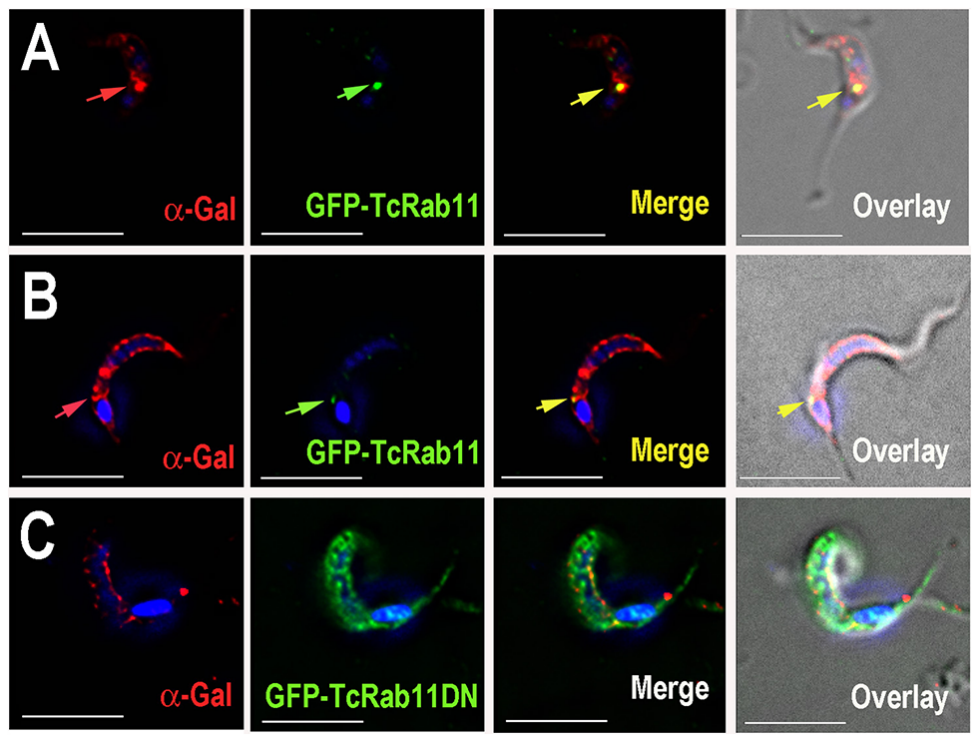
Localization of anti-Gal antibodies. (A) GFP-TcRab11 co-localizes with the anti-Gal antibodies in the CVC of the intermediate forms as detected by polyclonal antibody against GFP (*green arrow*) and anti-α-galactosyl antibodies from patients with chronic Chagas disease (*red arrow*), respectively. (B) Anti-Gal antibodies strongly label the surface of fully differentiated tissue culture derived trypomastigotes while GFP-TcRab11 labels the CVC. (C) GFP-TcRab11DN mutants show a punctated cytosolic localization (*green*) while anti-Gal antibodies (*red*) localize to the plasma membrane in intermediate stages. Scale bars (A–C) = 10 µm.

We next investigated whether other GPI-anchored proteins or integral membrane proteins required TcRab11 for trafficking to the surface. We selected for study TcTSSA II, which is a mucin-type GPI-anchored protein [42], and GPI-anchored mucin-like glycoproteins expressed on the cell surface of trypomastigotes that are recognized by anti-α-galactosyl antibodies from patients with chronic Chagas disease [43]–[45]. Also selected was a P-Type H^+^-ATPase, which is a proton pump important for maintenance of pH homeostasis and plasma membrane potential of *T. cruzi* different stages [61], [62] and that also localizes to the endocytic pathway of the parasites [63]. Antibodies against TcTSSA II co-localized with GFP-TcRab11 as assayed by indirect immunofluorescence analysis of intermediate forms (Fig. 8A) and intracellular amastigotes (Fig. 8B) and trafficked to the plasma membrane of trypomastigotes (Fig. 8C). Antibodies against α-Gal also co-localized with GFP-TcRab11 in the intermediate forms (Fig. 9A) before reaching the cell surface in the fully differentiated trypomastigotes (Fig. 9B). However, traffic of both mucins to the plasma membrane was not prevented in GFP-TcRab11DN-expressing parasites (Fig. 8D and 9C). Similarly, plasma membrane and intracellular localization of the P-type H^+^-ATPase, which did not co-localize with GFP-TcRab11, was not affected in GFP-Rab11DN parasites (Fig. 8E).

We also investigated the traffic of GPI-anchored surface antigens during metacyclogenesis, as described under Materials and Methods. We followed traffic of gp35/50 mucins, which are expressed in epimastigote and metacyclic forms. Immunofluorescence assays on GFP-TcRab11OE parasites with monoclonal antibody 2B10 [64] demonstrates the co-localization of GFP-TcRab11 with gp35/50 in the CVC of intermediate stages of differentiation (obtained at day 5 of metacyclogenesis) towards metacyclics trypomastigotes (Fig. S4A) and the lack of co-localization in metacyclic forms (obtained at day 10 of metacyclogenesis) (Fig. S4B). However, GFP-TcRab11DN mutants did not show any defect on the surface localization of this protein (Fig. S4C).

### CVC is enriched in lipid rafts

**Figure 10:**
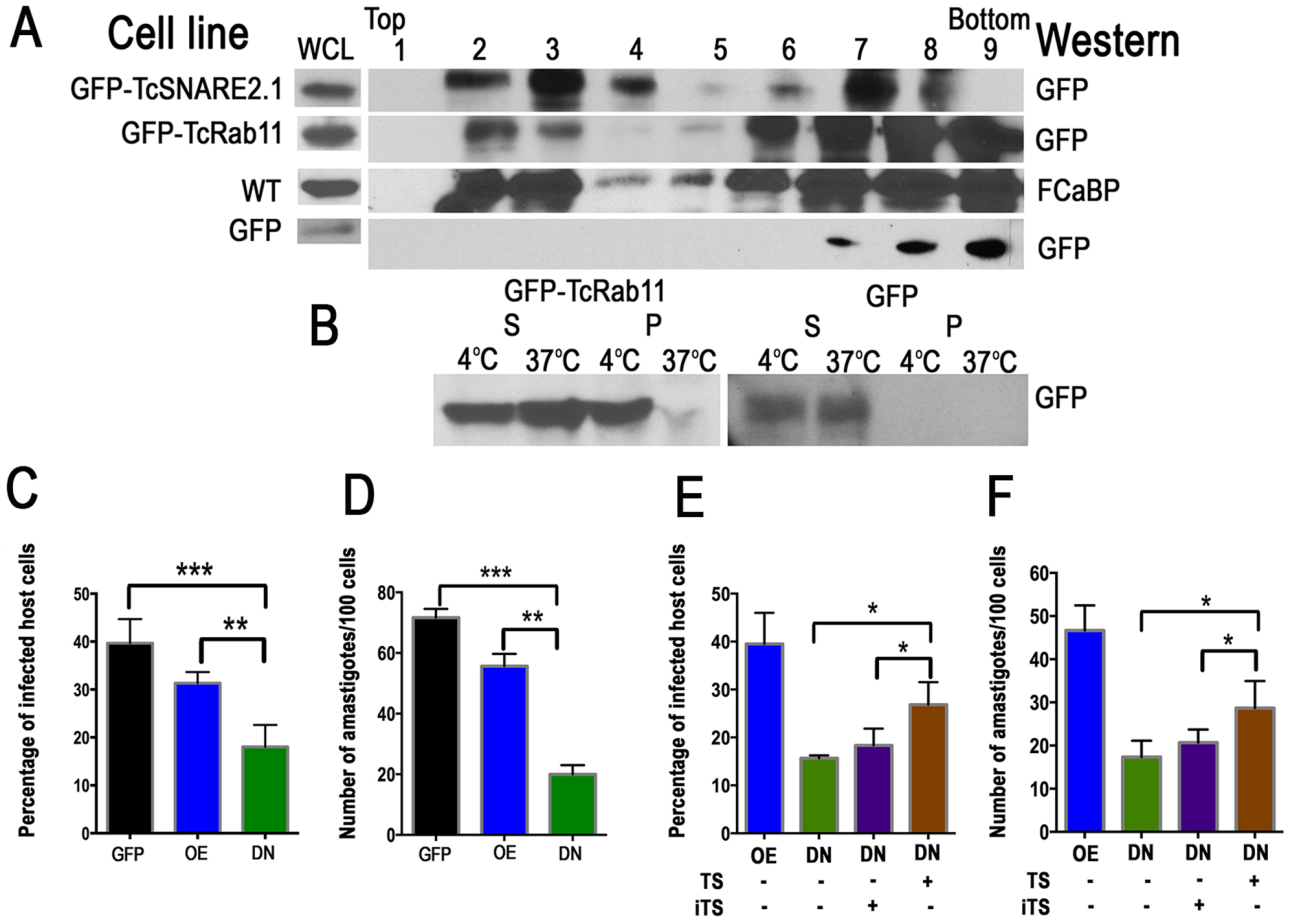
Association of CVC proteins with lipid rafts and reduced infectivity of TcRab11DN trypomastigote. (A) Parasite extracts were loaded at the bottom (fraction 9) of a discontinuous Optiprep density gradient and subjected to ultracentrifugation. Fractions were collected and analyzed by anti-GFP and anti-FCaBP immunoblotting. Fractions 2 and 3 contain the lipid raft interface. The TcSNARE2.1GFP (SNARE), GFP-TcRab11 (Rab11), and FCaBP floated to the lipid raft interface. Lanes 6–9 represent the heavier fractions of the GFP and FCaBP derivatives and GFP alone was detected in these fractions. A whole cell lysate (WCL) is included in each panel as a control of loading. Total protein in lysates of GFP-TcSNARE2.1-, GFP-TcRab11- and GFP-expressing epimastigotes were 1.41, 1.3 and 1.39 mg/ml, respectively. (B) *T. cruzi* expressing GFP-TcRab11 or GFP were solubilized in Triton X-100 at 4°C or 37°C and separated into soluble (S) and insoluble (P) fractions and analyzed by western blotting with anti-GFP. Rab11-GFP partitions in the pellet fraction at 4°C, but is solubilized at 37°C, whereas GFP is only detected in the soluble fraction. (C–D) Effect of TcRab11 overexpression (OE) or mutation (DN) on trypomastigote invasion of host cells. In vitro infection assays were carried out as described under Materials and Methods. (E–F) Partial rescue of the infectivity of DN trypomastigotes by their incubation in the presence of active TcTS and sialofetuin, whereas inactive *trans*-sialidase (iTS) does not rescue the infectivity of GFP-TcRab11DN mutants. Fetuin was present in all samples. Other conditions under Materials and Methods. Values in C–F are mean ± SD (n = 3). *, ** and *** indicate that differences are statistically significant compared with respective controls, *p*<0.05 (Ordinary one way ANOVA with Bonferroni post-test).

It has been proposed that GPI-anchored proteins acquire detergent resistance by fatty acid remodeling in the Golgi and their sorting is correlated with lipid raft formation at the *trans*-Golgi (TG) network [48]. To investigate whether the CVC possesses rafts we performed a detergent extraction of epimastigotes expressing different fusion constructs previously demonstrated to associate with this organelle (TcSNARE2.1-GFP that associates to the spongiome and GFP-TcRab11 that associates to the bladder [26], followed by density gradient centrifugation in an Optiprep gradient to isolate detergent-insoluble raft fractions. To determine whether rafts contained the fusion proteins, detergent-insoluble fractions were separated using SDS-PAGE and analyzed by western blotting with anti-GFP antibody. As a control for the isolation of lipid raft, a dually acylated protein that is highly enriched in the flagellar membrane of *T. cruzi*, a 24-kDa flagellar calcium-binding protein (FCaBP; [65]) was also used and detected with monoclonal antibodies. Fractions from *T. cruzi* epimastigotes expressing cytoplasmic GFP were used as negative control. Using this technique, we observed that GFP-TcSNARE2.1, GFP-TcRab11, and FCaBP floated to the top of the Optiprep gradient (Fig. 10A), suggesting the presence of lipid rafts in the CVC while GFP was associated with the heavier fractions. The association of GFP-TcRab11 with lipid rafts was further analyzed by another assay that is based on the temperature-dependence of lipid raft sensitivity to detergent [66] (Fig. 10B). As expected GFP-TcRab11 remained insoluble at 4°C and associated with the pellet fraction whereas it was soluble at 37°C after centrifugation, and a cytoplasmic protein, GFP, remained soluble at either temperature (Fig. 10B).

### *Trans*-sialidase activity requirement for infection

As TcTS is important for infectivity [36] we investigated whether GFP-TcRab11DN mutants were less effective than control cells or GFP-TcRab11OE parasites in the establishment of *T. cruzi* infections. Invasion was significantly reduced in GFP-TcRab11DN mutants as compared with controls transfected with GFP alone or GFP-TcRab11OE parasites (Figs. 10C and 10D). There was no significant difference between infections with wild-type trypomastigotes and trypomastigotes expressing GFP alone (Fig. S5A and S5B). Pre-incubation of GFP-TcRab11DN-expressing trypomastigotes for 30 min in the presence of recombinant TcTS and sialofetuin (as a donor of sialic acid) [37] (Fig. 10E and 10F), but not asialofetuin (Fig. S5C and S5D), partially rescued the infectivity of the parasites demonstrating the importance of TcTS activity for invasion of host cells.

The amino terminal 680 amino acids domain of TcTS contains the catalytic activity [67]. As a further control of the rescue experiments we did invasion experiments in the presence of inactive recombinant TcTS (iTS), whose crystal structure has been determined [68], and which differs in a single amino acid mutation (Tyr342His) that completely abolishes its TS activity, but retains its property to recognize terminal galactoses [32], [69]. The recombinant protein binds sialic acid and galactose *in vitro* [70],[71] and competes with a neutralizing antibody to a discontinuous epitope of TS [37] indicating that it is properly folded. Incubation in the presence of iTS did not rescue the infectivity of GFP-TcRab11DN mutants (Fig. 10E and 10F). All invasion assays were done in the absence of fetal bovine serum to prevent the presence of any other putative exogenous sialic acid donors.

## Discussion

The most significant finding of our studies is that GPI-anchored *trans*-sialidase (TcTS), mucins from tissue culture-derived or metacyclic trypomastigotes, and trypomastigote small surface antigen II (TcTSSA II) are trafficked to the plasma membrane of *T. cruzi* by an unconventional pathway involving the CVC and that the CVC is enriched in lipid rafts. We reported previously [26] that GFP-tagged TcRab11 localized to the CVC of epimastigotes of *T. cruzi*. We now confirmed those results using antibodies against the protein and found it in the CVC of different stages of the life cycle of the parasite. In contrast, dominant negative TcRab11 has a punctated cytosolic localization indicating that CVC localization is GTP-dependent. Expression of the dominant negative form of TcRab11 makes epimastigotes less responsive to hyposmotic and hyperosmotic stresses. These results, together with the detection by video microscopy of morphological changes in the CVC under different osmotic conditions further demonstrate the role of the CVC in both hyposmotic [4] and hyperosmotic [10] stresses. Expression of GFP-TcRab11DN prevents traffic of TcTS, but not of other GPI-anchored (TcTSSA II, mucins) or integral (H^+^-ATPase) membrane proteins to the plasma membrane of trypomastigotes, suggesting a specific role of TcRab11 in trafficking of TcTS, and that this is not a default pathway for all surface proteins. Dominant negative TcRab11 mutants might be acting by blocking or reducing the function of endogenous TcRab11, by competing or sequestering Rab11 effector proteins [49]. GFP-TcRab11DN-expressing trypomastigotes were less virulent but their pre-incubation with active, but not inactive, recombinant TcTS and a source of sialic acid partially rescued their virulence, underscoring the relevance of TcTS activity in infection. The identification of the specific role of TcTS in infection has been difficult to demonstrate in the past because of the impossibility of doing knockouts of the considerable number of gene copies encoding this protein scattered through the genome of this parasite.

In mammalian cells the GPI anchor is synthesized and transferred to proteins in the ER. GPI-anchored proteins (GPI-Aps) exit the ER from ER exit sites (ERES) and are transported to the Golgi complex in COPII-coated vesicles [48]. Acquisition of detergent resistance by fatty acid remodeling at the *trans*-Golgi facilitates their traffic to the plasma membrane [48]. A similar pathway has been proposed in case of the GPI-AP variant surface glycoprotein, or VSG, in *T. brucei*, with the peculiarity that VSG reaches first the flagellar pocket, which is the sole region for endo and exocytosis in this organism [72]. GPI-APs are selectively endocytosed by a unique pathway involving clathrin-independent vesicles in mammalian cells [48], while VSG is internalized via clathrin-coated vesicles in *T. brucei* [72]. VSG can be retrieved from early and late endosomes to the TbRab11-positive exocytic carriers and returned to the cell surface via the flagellar pocket [72].

Very little is known about GPI-AP secretion or endocytosis in *T. cruzi*, although uncoated vesicles containing transferrin have been observed budding off the flagellar pocket membrane and cytostome of epimastigotes [73]. The *trans*-sialidase family of proteins is predominantly expressed on the surface of trypomastigotes. Our results, using anti-TcTS antibodies, are consistent with the synthesis of *trans*-sialidase in amastigotes starting at least 48 h after infection [74] and its traffic through the CVC before reaching the surface at the flagellar pocket. Anti-TcTs (anti-SAPA) antibodies have been used before to localize TcTS to the surface of trypomastigotes by immunoelectron microscopy [58]. The presence of TcTS in the CVC by recycling from the surface is less probable because the protein is only detected in the plasma membrane at later time points and no further labeling of the CVC or endosomes is detected. It is possible that TcTS accumulates in the CVC when rapidly synthesized during conversion of amastigotes into trypomastigotes and then reaches a steady state and is below the limit of detection afterwards. In addition, it is known that TcTS is shed to the extracellular medium, including within the host cells [55], through the action of an endogenous phospholipase C, and also with vesicles of the plasma membrane [75]. Other GPI-APs like TcTSSA II, and other mucins, also traffic through the CVC before reaching the surface but its traffic to the surface is independent of TcRab11. A possible explanation for the traffic of GPI-anchored proteins through the CVC is that this organelle could be enriched in microdomains (or lipid rafts) in which lipids with straight lipid chains, such as glycosphingolipids, phospholipids, and palmitoylated proteins are packed together with cholesterol in a compact and stable fashion [76]. Our results support the presence of lipid rafts in the CVC of *T. cruzi*. In this regard, a proteomic analysis of GPI-anchored membrane protein fractions from epimastigotes and metacyclic trypomastigotes, extracted using the neutral detergent Triton X114 [77], detected several proteins that were later identified as present in the CVC [26], such as TcRab11, and the membrane proteins V-H^+^-ATPase, and V-H^+^-PPase.

Transfer of membrane [12], [16], [17], [21] and luminal [18], [20] proteins from the CVC to the plasma membrane has been reported before in several cells, including *T. cruzi* epimastigotes [21]. However, the mechanism involved was not known. In this work, we provide evidence for a role of TcRab11 in the transfer of TcTS to the surface of the infective stages of the parasite. The presence of vesicles labeled with antibodies against TcTS in the proximity of the plasma membrane suggests that vesicle trafficking from the CVC is involved in this process.

Rab proteins regulate a number of processes through their interactions with Rab effectors. Rab11 effectors in mammalian cells comprise myosin Vb, Sec15, a component of the exocyst complex, and a Family of Interacting Proteins or FIPs [52]. FIPS orthologues are absent in trypanosomes, as well as class V myosins but *T. brucei* Rab11 has been shown to interact with a Sec15 orthologue [78]. Interestingly both Rab11 [25] and Sec15 [79] localize to the CVC of *D. discoideum*, and it was suggested that the CVC of *D. discoideum* could be a precursor to the recycling endosomal system of other eukaryotes [2], [25].

Our results confirm the role of the CVC in both hyposmotic [4] and hyperosmotic [10] stress and suggest that TcRab11 is important for the response of these cells to these osmotic stresses. During its developmental cycle in the mammalian and insect hosts, *T. cruzi* faces critical environmental challenges and ones that are especially dramatic are the changes in osmolarity. Trypomastigotes need to resist osmolarities of 1,400 mOsm/kg and return to isosmotic conditions (300 mOsm/kg) when circulating through the renal medulla [80]. Amastigotes reproduce in some tissues that have higher osmolarity than serum (330 in lymphoid tissues vs 300 mOsm/kg) [81], and epimastigotes need to resist high osmolarities (∼1,000 mOsm/kg) in the rectal content of the insect vector [82]. TcRab11 appears to have a role in the resistance to these changes.

In summary, we describe a new unconventional pathway of GPI-APs to the plasma membrane that includes their traffic through the contractile vacuole complex. TcTS requires the participation of TcRab11 to reach the plasma membrane, while TcTSSA II and other mucins do not. This traffic of proteins through the CVC appears to be specific for GPI-APs, since other membrane proteins do not follow the same pathway.

## Materials and Methods

### Cell culture

Human foreskin fibroblasts (HFF) were grown in DMEM Low Glucose medium supplemented with 10% Cosmic Calf serum and 0.1% L-glutamine. Vero cells were grown in RPMI supplemented with 10% fetal bovine serum. L_6_E_9_ myoblasts were grown in DMEM High Glucose medium supplemented with 10% fetal bovine serum. Host cells were maintained at 37°C with 5% CO_2_. Tissue culture cell-derived trypomastigotes were obtained from L_6_E_9_ myoblasts infected with metacyclic trypomastigotes from stationary cultures of GFP-TcRab11OE and GFP-TcRab11DN parasites. *T. cruzi* amastigote and trypomastigote forms were collected from the culture medium of infected host cells, using a modification of the method of Schmatz and Murray [83] as described previously [84]. Epimastigotes from *T. cruzi* were cultured in liver infusion tryptose (LIT) medium containing 10% newborn serum at 28°C [10]. *T. cruzi* epimastigotes transfected with GFP-TcRab11OE and GFP-TcRab11DN were maintained in the presence of 250 µg/ml geneticin (G418).

### Chemicals and reagents

Fetal bovine serum, newborn calf serum, Dulbecco's phosphate buffer saline (PBS) and Hank's solution, 4′,6-diamidino-2-phenylindole (DAPI), DMEM and RPMI media, paraformaldehyde, bovine serum albumin, and protease inhibitors were purchased from Sigma (St. Louis, MO). Restriction enzymes, were from New England BioLabs (Ipswich, MA). pCR2.1-TOPO cloning kit, 1 kb plus DNA ladder, rabbit GFP antibodies and Gene Tailor Site-Directed Mutagenesis System were from Invitrogen (Life Technologies, Grand Island, NY). Hybond-N nylon membranes were obtained from PerkinElmer (Waltham, MA). TbRab11 purified antibodies were a gift from Mark Field (University of Dundee, Scotland). Monoclonal antibody 2B10 was a gift from Nobuko Yoshida (Federal University of São Paulo, Brazil), Chagasic α-Gal antibodies were a gift from Igor de Almeida (University of Texas, El Paso), antibody against TcTSSA II was a gift from Carlos Buscaglia (National University of San Martin, Argentina), monoclonal antibody FCaBP was a gift from David Engman (Northwestern University, Evanston, IL). Rabbit and goat GFP antibodies were from Abcam (Cambridge, MA). Recombinant active TcTS and inactive TcTS (iTS) were obtained as described [65]–[67]. BCA Protein Assay Reagent was from Pierce (Thermo Fisher Scientific, Rockford, IL). All other reagents were analytical grade. The oligonucleotides were ordered from Sigma or IDT (Coralville, IA).

### Metacyclogenesis

We followed the protocol described by Bourguignon et al. [85] with some modifications. Epimastigotes were obtained after 4 days in LIT medium and submitted to a stress (incubation for 2 h in a medium containing 190 mM NaCl, 17 mM KCl, 2 mM MgCl_2_, 2 mM CaCl_2_, 0.035% sodium bicarbonate, 8 mM phosphate, pH 6.9 at room temperature; triatome artificial urine (TAU) medium). After this stress, parasites were incubated for 96 h in TAU 3AAG medium (which consists of the previously described TAU medium supplemented with 10 mM L-proline, 50 mM sodium L-glutamate, 2 mM sodium L-aspartate, and 10 mM glucose). To increase the number of metacyclic forms, the contents of the flask were collected and resuspended in media containing fresh fetal bovine serum and incubated at 37°C for 20 h. The complement in the FBS kills epimastigotes while metacyclic trypomastigotes survive. Samples were harvested from the TAU 3AAG+FBS-containing medium at days 5 and 10 of cultivation.

### In vitro infection assay

HFF or irradiated myoblasts (6×10^5^ cells per well) were equally distributed in a 12-well plate on a sterile coverslip in their respective growth media (as mentioned above) and were incubated for 24 h at 37°C in a 5% CO_2_ atmosphere. The following day, the cells were washed once with Dulbecco's Hank's solution, and 6×10^6^ wild type, TcGFP, GFP-TcRab11OE, or GFP-TcRab11DN trypomastigotes were added to each well (10 trypomastigotes per myoblast or HFF), and they were incubated for 4 h at 37°C in a 5% CO_2_ atmosphere. To decrease the chances of contamination of cell derived-trypomastigotes with extracellular amastigotes, collections of parasites were centrifuged and incubated at 37°C for 2 h to allow trypomastigotes to swim to the surface. The supernatant was collected and used for subsequent invasion assays. Next, the parasites were removed from the plate, and the infected cells were washed extensively with Dulbecco's Hank's solution and fixed for immunofluorescence assays. For rescue experiments the same number of trypomastigotes were incubated with PBS, pH 7.4, in the absence of serum, and with fetuin or asialofetuin (solutions made in PBS, pH 7.4, and sterilized by filtration) at a final concentration of 10 µg/ml, and with 200 ng of active (TcTS) or inactive (iTS) *trans*-sialidase for 30 min at room temperature before infecting host cells. For attachment/internalization assays, recently internalized parasites, and parasites caught in the process of invasion, were considered and manually counted in at least 200 DAPI-stained cells in 3 independent experiments. The percentage of infected cells and the average number of parasites per infected cell were determined.

### Immunofluorescence and western blot analyses

For immunofluorescence microscopy, parasites were fixed in PBS, pH 7.4, with 4% paraformaldehyde, adhered to poly-lysine coverslips, and permeabilized for 3 min with PBS, pH 7.4, containing 0.3% Triton X-100. Permeabilized cells were quenched for 30 min at room temperature with 50 mM NH_4_Cl and blocked overnight with 3% BSA in PBS, pH 8.0. Both primary and secondary antibodies were incubated for 1 h at room temperature. Coverslips were mounted by using a mounting medium containing DAPI at 5 µg/ml for staining DNA-containing organelles. For imaging of intracellular parasites, mammalian cells were seeded onto sterile coverslips in 12-well culture plates and allowed to grow for 24 h. To semi-synchronize the infection, we added the parasites at a ratio of 10∶1 (parasite/host cell) for 4 hours, washed the cells to eliminate extracellular parasites and fixed in cold methanol for 30 min. Infected cells were prepared for immunofluorescence analyses as described above for extracellular parasites, except for the permeabilization that was performed for 10 min with Triton X-100 in PBS, pH 7.4. The dilution used for primary antibodies were as follows: rabbit anti-TcAQP1, 1∶50 [3]; rabbit anti-TbRab11 [24] 1∶200; rabbit polyclonal anti-GFP, 1∶500; rabbit anti-TcTS [57], 1∶2,000; rabbit anti-TcTSSA II [42], 1∶200; rabbit anti-H^+^ATPase [63], 1∶100. Differential interference contrast (DIC) and direct fluorescence images were obtained by using an Olympus IX-71 inverted fluorescence microscope with a PhotometrixCoolSnapHQ charge-coupled device camera driven by Delta Vision softWoRx3.5.1 (Applied Precision, Issaquah, WA). Images were deconvolved for 10 cycles using the same software and applying the “noise filter” at “medium” mode. This is an automatic deconvolution software and was applied to all channels; brightness and contrast were the same in all channels. The figures were built by using Adobe Photoshop 10.0.1 (Adobe System, Inc., San Jose, CA).

For western blot analysis, ∼10^8^ *T. cruzi* epimastigotes, amastigotes or trypomastigotes were collected by centrifugation at 1,600× *g* for 10 min, washed twice in PBS, pH 7.4, and resuspended in modified radioimmunoprecipitation analysis (RIPA) buffer (150 mM NaCl, 20 mM Tris-Cl pH 7.5, 1 mM EDTA, 1% SDS and 0.1% Triton X-100) containing protease inhibitor cocktail (2 mM EDTA, 2 mM phenylmethylsulfonyl fluoride (PMSF), 2 mM tosylphenylalanylchloromethyl ketone (TPCK), 0.1 mM *trans*-epoxysuccinyl-L-leucylamido(4-guanidino) butane (E64) and Sigma P8340 protease inhibitor cocktail, 1∶250). Cells were mechanically fragmented by passing lysates through a 20-gauge needle five times. The protein concentration was estimated by spectrophotometry, using the BCA Protein Assay Reagent. Twenty micrograms of protein from each total cell lysate was mixed with 2X Laemmli sample buffer, boiled for 5 min, and total homogenate of each sample were separated by SDS-PAGE. Proteins were transferred onto nitrocellulose membranes and blocked overnight with 5% nonfat dry milk in PBS-0.1% Tween 20 (PBS-T). The following primary antibodies were applied at room temperature for 1 hr: rabbit anti-GFP at 1∶1000, mFCaBP at 1∶50, and rabbit anti-TcTS at 1∶5000. Densitometric analysis of 3 independent experiments was performed with Alfa-Imager software.

### Flow cytometry

Tissue culture-derived trypomastigotes (10^6^ cells) were fixed in 4% paraformaldehyde in PBS, pH 7.4, and washed in blocking solution (3% BSA in PBS). After washing, cells were incubated with the anti-TcTS (1∶2,000 dilution) in blocking solution for 1 hr on ice. Parasites were washed and incubated in Alexa Fluor 633 goat anti-rabbit for one hour on ice. After washing, parasites were resuspended in PBS and samples were sorted on a MoFlo cytometer (Cytomation, Fort Collins, CO) using a 633 nm argon laser for excitation and an emission filter of 632/647 nm band pass. Samples were manually gated to eliminate debris and dead parasites or cells. Data were analyzed using Summit version 3.1 (Cytomation) and prepared for publication using Flowjo version 4.0.2 (Treestar, San Carlos, CA).

### Generation of TcRab11 dominant negative mutant and transfection

Dominant negative forms of Rab11 were constructed via site directed mutagenesis by the use of Gene Tailor Site-Directed Mutagenesis System. This method involved methylating the TOPO blunt end vector containing the coding sequence for TcRab11 with DNA methylase at 37°C for 1 hour, followed by amplification of the plasmid in a mutagenesis reaction with two overlapping primers, forward, 5′-GCGATAGTGGCGTCGGCAAGAACAACCTCATGACG-3′ and reverse, 5′-CTTGCCGACGCCACTATCGCCGATGATGACAAC-3′ of which the forward primer had the target mutation, resulting in the mutation of amino acid serine to asparagine. Mutations were confirmed by sequencing (Yale DNA Analysis Facility, Yale University, New Haven, Connecticut). After transformation the resulting plasmid TcRab11S21N in TOPO was digested with restriction enzymes BamHI and HindIII. The circular pTEX-N-GFP vector was linearized by the corresponding restriction enzymes. Finally, TcRab11S21N insert was ligated to pTEX-N-GFP followed by transformation. The plasmid pTEX-N-GFPTcRab11S21N was sequenced to confirm that the correct reading frame was used. *T. cruzi* CL strain epimastigotes were transfected in cytomix (120 mM KCl, 0.15 mM CaCl_2_, 10 mM K_2_HPO_4_, 2 mM EDTA, 5 mM MgCl_2_, pH 7.6) containing 50 µg of the plasmid construct in a 4 mm cuvette. The cuvette was cooled on ice for 10 min and pulsed 3 times (1.5 kV, 25 µF) with a Gene Pulser Xcell (Bio-Rad), and expression of GFP-fusion proteins was verified by western blot analyses. Stable cell lines were established under drug selection with G418 at 250 µg/ml. Enrichment of GFP fluorescent parasites was performed with a high-speed cell sorter when needed (MoFlo Legacy; Beckman-Coulter, Hialeah, FL).

### Cryo-immunoelectron microscopy

HFF containing intracellular GFP-TcRab11OE expressing amastigotes were detached by treating the T25 flasks with 0.25% trypsin at 96 h and 106 h post-infection. The contents of the flask were collected and amastigotes were isolated from the host cells by passing them through a 20-gauge needle. The released amastigotes (with ∼5% contamination of trypomastigotes) were fixed in 4% paraformaldehyde in 0.1 M cacodylate buffer, pH 7.3 for 1 h on ice. Epimastigotes were collected as described above and submitted to hyposmotic conditions. Hyposmotic stress was induced by addition of hyposmotic buffer (64 mM NaCl, 4 mM KCl, 1.8 mM CaCl_2_, 0.53 mM MgCl_2_, 5.5 mM glucose, 50 mM D-mannitol, 5 mM Hepes-Na, pH 7.4) to a final osmolarity of 177 mosmol/L for 2 min and then fixed with 0.1% glutaraldehyde and 4% paraformaldehyde in 0.1 M cacodylate buffer, pH 7.3 for 1 h on ice. The samples were processed for cryo-immunoelectron microscopy at the Molecular Microbiology Imaging Facility, Washington University School of Medicine. The antibodies used were: goat anti-GFP (1∶500), rabbit anti-GFP (1∶50), rabbit anti-TcTS (1∶250), donkey anti-goat 18 nm colloidal gold, donkey anti-rabbit 18 nm colloidal gold, donkey anti-rabbit 12 nm colloidal gold.

### Cell volume measurements

*T. cruzi* epimastigotes (GFP-TcRab11OE, GFP-TcRab11DN and wild-type) at log phase of growth (3 days) were collected at 1,600 g for 10 min (at a density of 1×10^8^/ml), washed twice in PBS and resuspended in isosmotic buffer (64 mM NaCl, 4 mM KCl, 1.8 mM CaCl_2_, 0.53 mM MgCl_2_, 5.5 mM glucose, 150 mM D-mannitol, 5 mM Hepes-Na, pH 7.4, to a final osmolarity of 282 mosmol/L, as determined using an Advanced Instruments 3D3 osmometer. Relative cell volume changes after osmotic stress were measured by light scattering. Aliquots of parasites were distributed in 96 well plates such that each well had 1×10^7^ cells and an appropriate volume of the corresponding buffer was added for osmotic stress. Hyposmotic stress was induced by dilution of the isosmotic cell suspension with deionized water to a final osmolarity of 150 mOsm at time zero. Hyperosmotic stress was induced by addition of hyperosmotic buffer (64 mM NaCl, 4 mM KCl, 1.8 mM CaCl_2_, 0.53 mM MgCl_2_, 5.5 mM glucose, 500 mM D-mannitol, 5 mM Hepes-Na, pH 7.4) to a final osmolarity of 650 mosmol/L. Absorbance at 550 nm was monitored every 10 sec for 10 min using a SpectraMax M2^e^ plate reader (Molecular Devices) [10]. A decrease in absorbance corresponds to an increase in cell volume. The results were normalized respect to the value of a 3 min pre-reading under isosmotic conditions.

### Video microscopy

Epimastigotes (1×10^8^ cells) in logarithmic phase of growth were collected by centrifugation, washed 3 times in PBS and resuspended in isosmotic buffer (composition mentioned above). GFP-TcRab11 overexpressing epimastigotes were immobilized with poly-L-lysine on coverslips in MatTek glass bottom dishes for 30 min at room temperature. Unattached cells were washed with PBS. To induce hyposmotic stress the isosmotic buffer was diluted by 1∶1 with deionized water. Hyperosmotic stress was induced by bathing the chamber with hyperosmotic buffer (as described above). Time lapse photographic data were collected at 1 sec intervals with a 60× objective and a 1024×1024 field with a Delta Vision Elite system (Applied Precision). Video sequences were reconstructed using Quicktime software.

### Lipid raft isolation

An Optiprep gradient centrifugation (sucrose float) procedure was used to isolate lipid rafts from *T. cruzi* epimastigotes wild type Y strain and those expressing GFP, GFP-TcRab11 and GFP-TcSNARE2.1 fusion proteins using lysates equivalent to 2.5×10^8^ mid log phase epimastigotes for each sample. The procedure was as described before [86] with minor modifications. Briefly, tubes were centrifuged continuously at 4°C in a Beckman Coulter Optima L-100XP ultracentrifuge with a Beckman SW32Ti rotor at 35,000 rpm (210,000× *g*) for 5 h and then 25,000 rpm (107,000× *g*) for 8 h. After collecting the fractions, a 24 µl aliquot of each fraction was mixed with 6 µl of 5X SDS-PAGE loading buffer, boiled for 10 min, and processed for SDS-PAGE and western blot analysis as above. The procedure for temperature-dependent Triton X-100 extraction for GFP-TcRab11- and GFP-expressing epimastigotes was as described [66].

## Supporting Information

Figure S1 **Cryo-immunogold electron microscopy localization of GFP-TcRab11 in epimastigotes.** (A–D) show different views of the CVC. Epimastigotes were isolated and submitted to hyposmotic stress as described under Materials and Methods. GFP-TcRab11 was detected with rabbit anti-GFP, and donkey anti-rabbit 18 nm colloidal gold. GFP-TcRab11 localizes mainly to the CV bladder. Arrows in C show labeling of the dilated spongiome (Sp) tubules. CV: contractile vacuole bladder; Sp: spongiome; Fl, flagellum; K, kinetoplast. Scale bars = 100 nm.
(TIF)

Figure S2 **Growth rate, and western blot analyses of overexpressed GFP-TcRab11.** (A) Growth rate of epimastigotes overexpressing (OE, *blue*) or expressing the dominant negative (DN, *green*) mutant of GFP-TcRab11, as compared to controls (C, *red*). (B) Western blot analyses of GFP-TcRab11OE (OE), GFP-TcRab11DN (DN) and GFP-expressing (GFP) epimastigotes. Membranes were stripped and re-incubated with anti-tubulin antibody as a loading control (*bottom panel*). (C) Densitometry analysis of western blots of lysates from GFP-TcRab11 overexpressing epimastigotes (OE) as compared to those of control cells. Values in arbitrary units (AU) correspond to mean ± SD from 3 independent experiments. (D) Western blot analyses of GFP-TcRab11OE (OE), GFP-TcRab11DN (DN) and GFP-expressing (GFP) trypomastigotes. Membranes were stripped and re-incubated with anti-tubulin antibody as a loading control (*bottom panel*).
(TIF)

Figure S3 **TcAQP1 localization is not affected in GFP-TcRab11DN mutants and western blot analysis of wild type and GFP-TcRab11DN shows specificity of anti-TcTS antibodies.** (A) Co-localization of GFP-TcRab11, as detected with antibodies against GFP (*green arrow*), with antibodies against TcAQP1 (α-TcAQP, *red arrow*) in epimastigotes. (B) GFP-TcRab11DN mutants show a punctated cytosolic localization as detected with anti-GFP (*green*), while antibodies against TcAQP1 still localize to the CVC (*red arrows*). Co-localization is indicated in Merge images (*yellow and red arrows*). Bars = 10 µm. (C) Western blot analyses of GFP-TcRab11DN (DN), and wild type (WT) trypomastigotes using anti-TcTS antibodies. Membranes were stripped and re-incubated with anti-tubulin antibody as a loading control (tubulin, *bottom panel*).
(TIF)

Figure S4 **Localization of GFP-TcRab11 and gp35/50 mucins during metacyclogenesis.** (A) GFP-TcRab11 co-localizes with gp35/50 mucins in the CVC of intermediate forms, as detected with polyclonal antibody against GFP (*green arrow*), and monoclonal antibody 2B10 (*red arrow*), respectively. Surface localization of gp35/50 is also evident (*red*). (B) GFP-TcRab11 (*green arrows*) does not co-localize with gp35/50 mucins, which have a surface localization in metacyclic trypomastigotes (*red*). (C) GFP-TcRab11DN mutants show a punctated cytosolic localization of TcRab11DN (*green*) while gp35/50 mucins (*red*) localize to the plasma membrane in intermediate stages. Scale bars (A–C) = 10 µm.
(TIF)

Figure S5 **Infections of host cells by trypomastigotes overexpressing GFP-TcRab11.** (A–B) GFP-TcRab11 overexpression (OE) does not cause significant changes in trypomastigote invasion of host cells as compared to wild type trypomastigotes. In vitro infection assays were carried out as described under Materials and Methods. (C–D). Recombinant active *trans*-sialidase rescues the infectivity of GFP-TcRab11DN mutants in the presence of fetuin (F) but not in the presence of asialofetuin (A). Other conditions were as under Materials and Methods.
(TIF)

Video S1 **Changes in the contractile vacuole complex under hyposmotic conditions.**
(MOV)

Video S2 **Changes in the contractile vacuole complex under hyperosmotic conditions.**
(MOV)
