# Supplementary figures and images for: Rab11 Regulates Trafficking of Trans-sialidase to the Plasma Membrane through the Contractile Vacuole Complex of Trypanosoma cruzi
Source: PLoS Pathog. 2014 Jun 26;10(6):e1004224. doi: 10.1371/journal.ppat.1004224 (PMC4072791; doi:10.1371/journal.ppat.1004224)

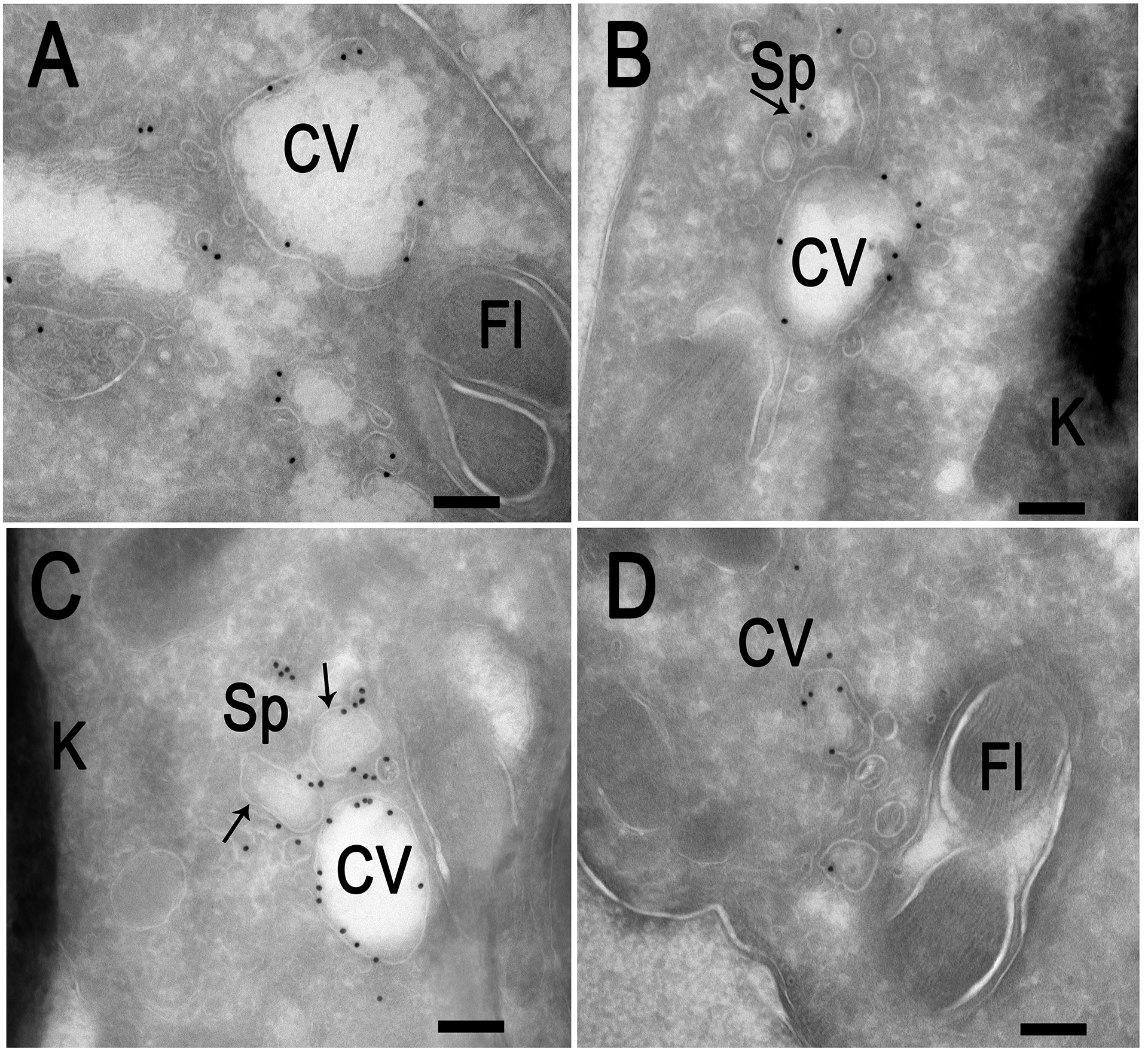

Supplement: Figure S1 — Cryo-immunogold electron microscopy localization of GFP-TcRab11 in epimastigotes. (A–D) show different views of the CVC. Epimastigotes were isolated and submitted to hyposmotic stress as described under Materials and Methods. GFP-TcRab11 was detected with rabbit anti-GFP, and donkey anti-rabbit 18 nm colloidal gold. GFP-TcRab11 localizes mainly to the CV bladder. Arrows in C show labeling of the dilated spongiome (Sp) tubules. CV: contractile vacuole bladder; Sp: spongiome; Fl, flagellum; K, kinetoplast. Scale bars = 100 nm. (TIF) [file ppat.1004224.s001.tif]

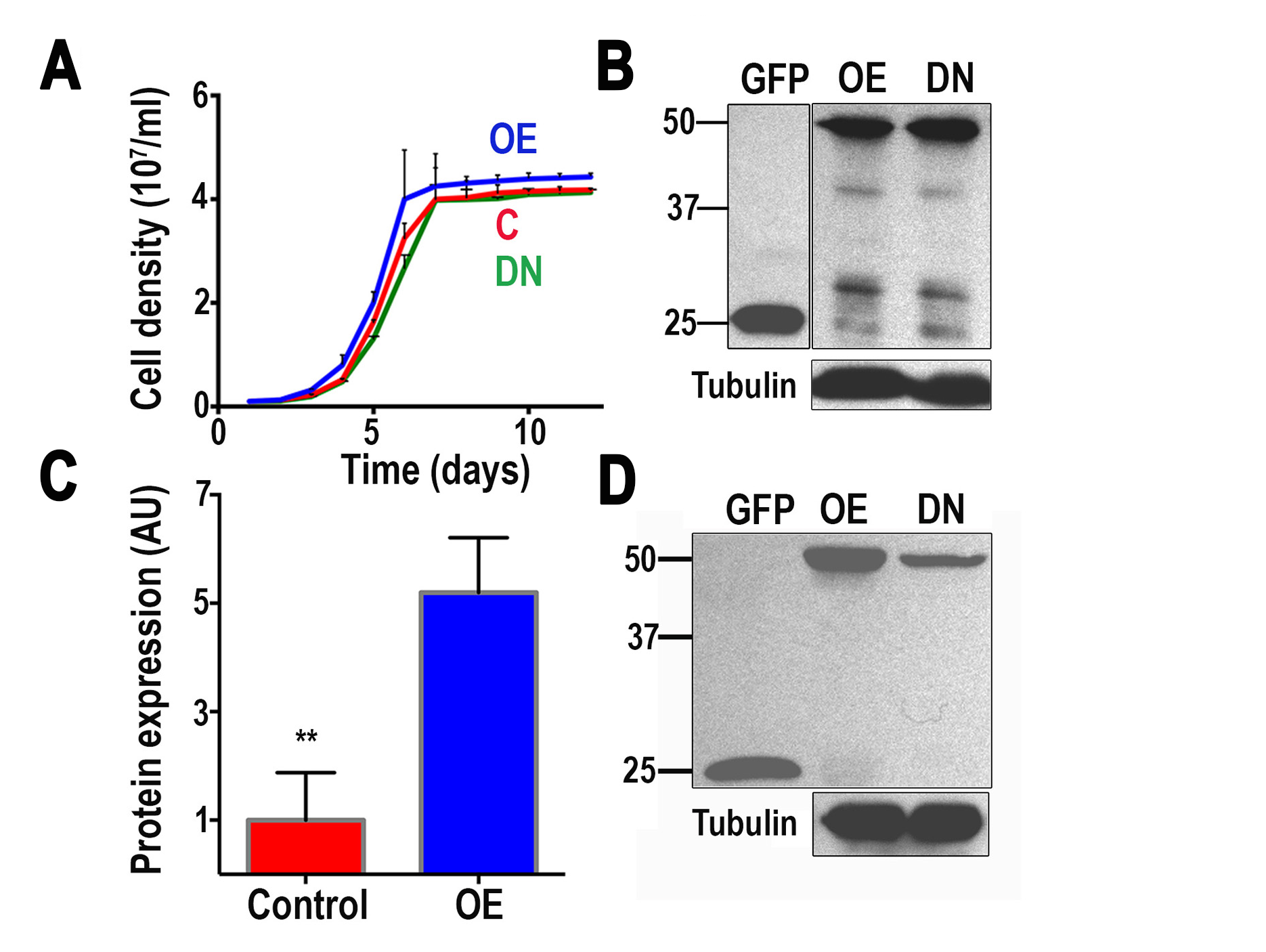

Supplement: Figure S2 — Growth rate, and western blot analyses of overexpressed GFP-TcRab11. (A) Growth rate of epimastigotes overexpressing (OE, blue) or expressing the dominant negative (DN, green) mutant of GFP-TcRab11, as compared to controls (C, red). (B) Western blot analyses of GFP-TcRab11OE (OE), GFP-TcRab11DN (DN) and GFP-expressing (GFP) epimastigotes. Membranes were stripped and re-incubated with anti-tubulin antibody as a loading control (bottom panel). (C) Densitometry analysis of western blots of lysates from GFP-TcRab11 overexpressing epimastigotes (OE) as compared to those of control cells. Values in arbitrary units (AU) correspond to mean ± SD from 3 independent experiments. (D) Western blot analyses of GFP-TcRab11OE (OE), GFP-TcRab11DN (DN) and GFP-expressing (GFP) trypomastigotes. Membranes were stripped and re-incubated with anti-tubulin antibody as a loading control (bottom panel). (TIF) [file ppat.1004224.s002.tif]

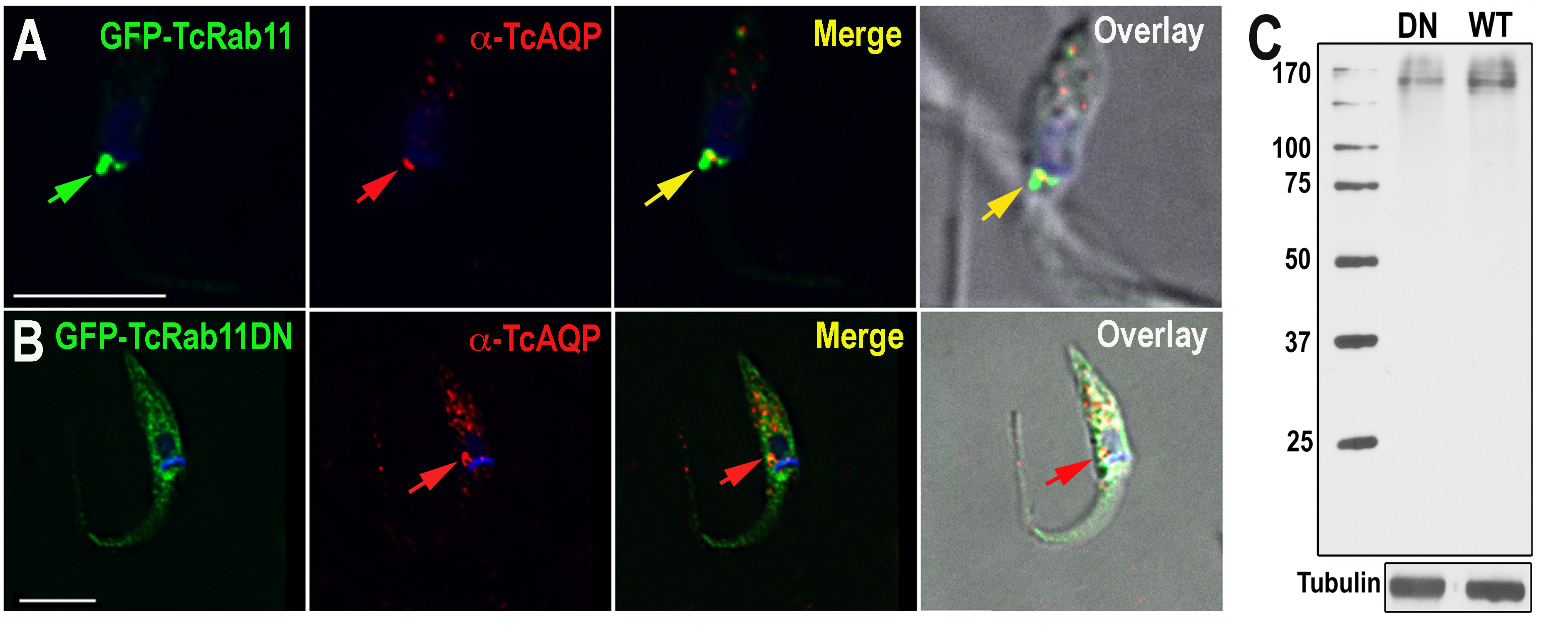

Supplement: Figure S3 — TcAQP1 localization is not affected in GFP-TcRab11DN mutants and western blot analysis of wild type and GFP-TcRab11DN shows specificity of anti-TcTS antibodies. (A) Co-localization of GFP-TcRab11, as detected with antibodies against GFP (green arrow), with antibodies against TcAQP1 (α-TcAQP, red arrow) in epimastigotes. (B) GFP-TcRab11DN mutants show a punctated cytosolic localization as detected with anti-GFP (green), while antibodies against TcAQP1 still localize to the CVC (red arrows). Co-localization is indicated in Merge images (yellow and red arrows). Bars = 10 µm. (C) Western blot analyses of GFP-TcRab11DN (DN), and wild type (WT) trypomastigotes using anti-TcTS antibodies. Membranes were stripped and re-incubated with anti-tubulin antibody as a loading control (tubulin, bottom panel). (TIF) [file ppat.1004224.s003.tif]

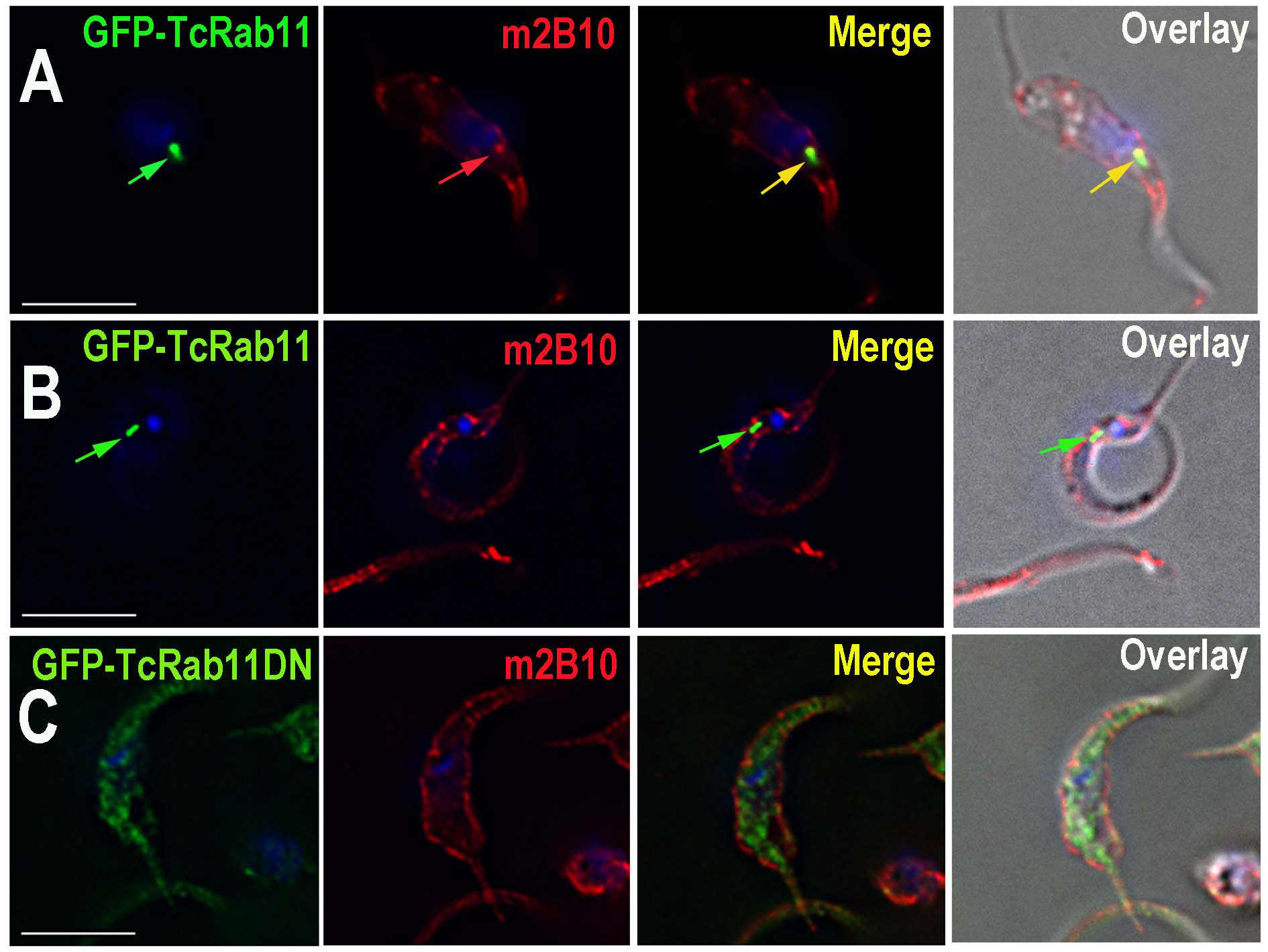

Supplement: Figure S4 — Localization of GFP-TcRab11 and gp35/50 mucins during metacyclogenesis. (A) GFP-TcRab11 co-localizes with gp35/50 mucins in the CVC of intermediate forms, as detected with polyclonal antibody against GFP (green arrow), and monoclonal antibody 2B10 (red arrow), respectively. Surface localization of gp35/50 is also evident (red). (B) GFP-TcRab11 (green arrows) does not co-localize with gp35/50 mucins, which have a surface localization in metacyclic trypomastigotes (red). (C) GFP-TcRab11DN mutants show a punctated cytosolic localization of TcRab11DN (green) while gp35/50 mucins (red) localize to the plasma membrane in intermediate stages. Scale bars (A–C) = 10 µm. (TIF) [file ppat.1004224.s004.tif]

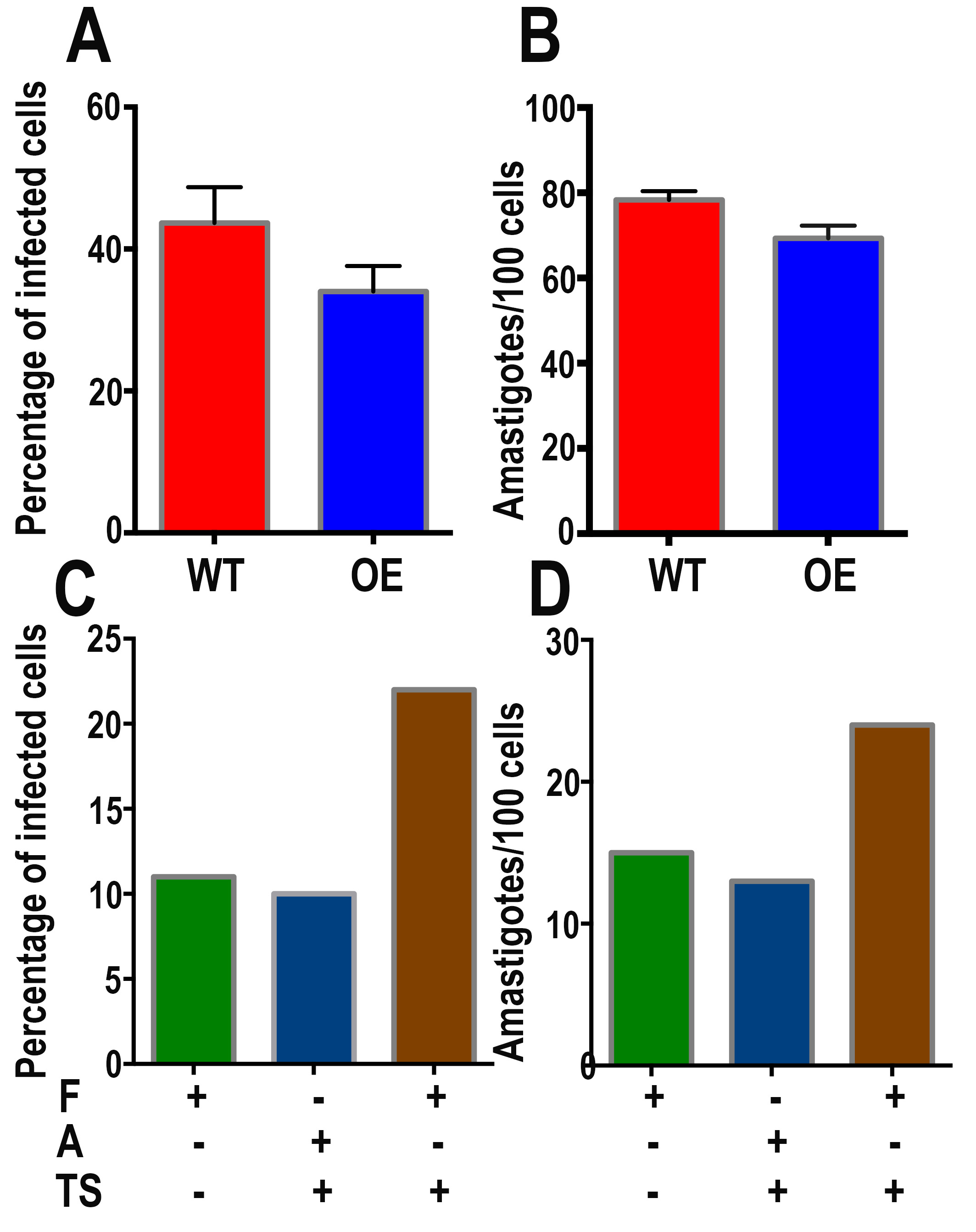

Supplement: Figure S5 — Infections of host cells by trypomastigotes overexpressing GFP-TcRab11. (A–B) GFP-TcRab11 overexpression (OE) does not cause significant changes in trypomastigote invasion of host cells as compared to wild type trypomastigotes. In vitro infection assays were carried out as described under Materials and Methods. (C–D). Recombinant active trans-sialidase rescues the infectivity of GFP-TcRab11DN mutants in the presence of fetuin (F) but not in the presence of asialofetuin (A). Other conditions were as under Materials and Methods. (TIF) [file ppat.1004224.s005.tif]
